# The Benefits of Psychosocial Interventions for Mental Health in People Living with HIV: A Systematic Review and Meta-analysis

**DOI:** 10.1007/s10461-017-1757-y

**Published:** 2017-03-30

**Authors:** Sanne van Luenen, Nadia Garnefski, Philip Spinhoven, Pascalle Spaan, Elise Dusseldorp, Vivian Kraaij

**Affiliations:** 1 Section of Clinical Psychology, Institute of Psychology, Faculty of Social and Behavioural Sciences, Leiden University, P.O. Box 9555, 2300 RB Leiden, The Netherlands; 2 Department of Psychiatry, Leiden University Medical Center, Leiden, The Netherlands; 3 Section of Methodology and Statistics, Institute of Psychology, Leiden University, Leiden, The Netherlands

**Keywords:** HIV, Psychosocial intervention, Mental health, Depression, Meta-analysis

## Abstract

In this systematic review and meta-analysis we investigated the effectiveness of different psychosocial treatments for people living with HIV (PLWH) and mental health problems. Additionally, characteristics that may influence the effectiveness of a treatment (e.g., treatment duration) were studied. PubMed, PsycINFO and Embase were searched for randomized controlled trials on psychosocial interventions for PLWH. Depression, anxiety, quality of life, and psychological well-being were investigated as treatment outcome measures. Sixty-two studies were included in the meta-analysis. It was found that psychosocial interventions for PLWH had a small positive effect on mental health (*ĝ* = 0.19, 95% CI [0.13, 0.25]). Furthermore, there was evidence for publication bias. Six characteristics influenced the effectiveness of a treatment for depression. For example, larger effects were found for studies with psychologists as treatment providers. To conclude, this systematic review and meta-analysis suggests that psychosocial interventions have a beneficial effect for PLWH with mental health problems.

## Resumen

En esta revisión sistemática y meta-análisis se ha investigado la efectividad de los diferentes tratamientos psicosociales para las personas viviendo con VIH (PVVIH) y con problemas de salud mental. Adicionalmente, hemos estudiado las características que pueden influir en la efectividad de un tratamiento (por ejemplo, duración del tratamiento). Como medidas de tratamiento, pruebas controladas aleatorizadas de las intevenciones psicosociales para depresión, ansiedad, calidad de vida y bienestar psicológico fueron buscadas en PubMed, PsycINFO y Embase. Sesenta y dos estudios se han incluido en el metanálisis. Hemos encontrado que intervenciones psicosociales para las PVVIH tuvieron un menor efecto positivo en la salud mental (*ĝ* = 0.19, IC del 95% [0.13, 0.25]). Además, hubo evidencia de sesgo en la publicación. Seis características influyeron la efectividad del tratamiento para la depresión. Por ejemplo, hemos encontrado efectos mayores en estudios con psicólogos como proveedores del tratamiento. Concluyendo, esta revisión sistemática y meta-análisis indica que las intervenciones psicosociales tienen un efecto beneficioso para gente con PVVIH y con problemas de salud mental.

## Introduction

In recent decades, due to the use of antiretroviral medication, HIV has become a chronic illness instead of a disease that rapidly leads to severe sickness and death. However, people living with HIV (PLWH) may still suffer from physical symptoms, such as pain and lack of energy [1]. In addition, mental health problems such as depression and anxiety are common among PLWH [2–4]. In PLWH, the prevalence rate of mood disorders or clinically significant depressive symptoms is approximately 33% [5], and the prevalence rate of anxiety disorders about 20% [6]. Several psychosocial factors—such as isolation, stigma, discrimination, lack of support, and drug abuse—can contribute to feelings of depression and anxiety [4, 7]. In turn, mental health problems may have various negative consequences for PLWH. For example, it has been shown that PLWH with depressive symptoms have a higher risk of poor adherence to antiretroviral therapy (ART); they are also more at risk for HIV-related morbidity and mortality [8]. More specifically, in PLWH psychological factors (such as depressive symptoms and stress) have been shown to be related to immune measures, such as decreased cluster of differentiation 4 (CD4) cell count and increased viral load [9, 10]. In view of the above research, it seems apparent that depression and anxiety in PLWH may decrease well-being and quality of life [11].

Given the impact that psychological symptoms have on the health and well-being of PLWH, it is very important to treat these symptoms. Several psychosocial interventions, such as cognitive behavioral therapy (CBT), supportive interventions, meditation, and stress management interventions, have been developed for PLWH with mental disorders. Various reviews and meta-analyses have found that these interventions are effective in reducing symptoms of depression, anxiety, and distress in PLWH [12–18]. The format of these interventions may differ from a group-based or individualized format to interventions where the PLWH’s partner or other family members are also involved. In addition to assessing the overall effectiveness of various psychosocial interventions, it is also important to compare interventions with each other and determine whether specific interventions stand out as the most effective to improve mental health in PLWH. With this information, more targeted treatment may be provided in the future. The interventions that seem to be the most effective could be offered first, which might improve the psychological care of PLWH. However, previous meta-analyses and reviews [12, 13, 15–18] have not compared interventions with each other to discover which specific psychosocial interventions are the most effective for PLWH with mental health problems.

Additionally, it is also meaningful to investigate whether certain characteristics may influence the effectiveness of the treatment. We can distinguish two types of characteristics that may act as moderators. The first type are characteristics of the intervention, such as treatment duration, intervention techniques, or the provider of the therapy. If we have more knowledge about the effect of treatment characteristics on the effectiveness of an intervention, this can be used to improve existing interventions by including the most beneficial aspects when designing new interventions for PLWH with mental health problems. If we find, for example, that interventions provided online or by a psychologist are more effective than interventions provided by others (e.g., peers), it may be useful to design new interventions that are provided online or by psychologists. The second type of possible moderators are characteristics of the study, such as the sample that was used or the type of control group. These characteristics may partly explain why some studies find larger effects than others. For instance, it may be that studies with many female participants show larger effects than studies with many males. If this is the case, it may be useful to consider this aspect in future studies. As yet, not much research is available about moderators of treatment effect in PLWH. However, moderating factors were taken into account in one meta-analysis, which found that stress-management interventions for PLWH reduced anxiety symptoms. In this research, the effect of the intervention was found to be larger when they included more women, more participants with anxiety symptoms at baseline, younger participants, and made less use of medication adherence information and/or planning in an intervention [16].

Most previous meta-analyses have focused on particular psychological interventions (e.g., CBT or meditation) and mostly also on a particular outcome (such as alleviation of depression or anxiety) [12–18]. However, no meta-analysis has yet investigated which psychosocial interventions are the most effective on psychological outcomes. The first aim of the present systematic review and meta-analysis was to investigate this. This meta-analysis included various psychosocial interventions for PLWH, including CBT, support interventions (e.g., peer support), interpersonal psychotherapy, stress management, mindfulness, coping improvement interventions, and family interventions. In addition, multiple outcomes were studied: depression, anxiety, quality of life, and psychological well-being. Only randomized controlled trials (RCTs) were included in the meta-analysis. As it is important to investigate moderators of intervention effect, and this was not examined in detail in previous meta-analyses, the second aim of the current meta-analysis was to carry out a moderator analysis. Since we included a lot of different intervention types and outcomes, we expected that there would be large differences between study effect sizes. A moderator analysis could give more information about which characteristics might explain these differences in results. Various possible moderators were included: intervention characteristics such as intervention duration, therapy provider, and intervention techniques (e.g., stress-management); and study characteristics such as attrition, study quality, and sample characteristics. To conclude: we investigated and provide an overview of the effectiveness for PLWH of psychosocial interventions in decreasing depression and anxiety and improving quality of life and psychological well-being. In addition, we investigated whether specific interventions stood out as having the greatest effect on these outcomes and we studied moderators of intervention effect.

## Methods

### Search Strategy and Study Selection

Several strategies were used to search for relevant RCTs. First, we searched in the electronic databases PubMed, PsycINFO, and Embase on September 29, 2014. Search words included terms related to HIV/AIDS, various types of psychosocial interventions (e.g., CBT, psychotherapy), and outcomes (e.g., depression, quality of life). The search strategy with keywords can be found in the Appendix. Second, we also searched for papers in the references of available meta-analyses and reviews about the subject.

Studies were included when they met all of the following criteria: (1) RCT; (2) evaluation of a psychosocial intervention (see definition below); (3) participants in the study are HIV positive and are 18 years or older; (4) year of data collection is later than 1995 (see explanation below); (5) the outcome variables that were studied belong to one or more of the following categories: depression, anxiety, psychological well-being, or quality of life; (6) studies were written in the English language; and (7) data to calculate effect sizes was present in the paper or retrieved from the authors. Regarding the second inclusion criterion, a psychosocial intervention was operationalized as an intervention that aimed to change thoughts, emotions and/or behavior of PLWH and had a psychosocial component. Therefore, physical interventions (such as exercise), were not included in the meta-analysis. Regarding the fourth inclusion criterion, we decided to include studies that collected data later than 1995, since antiretroviral medication was developed in 1996 and consequently the future prospects of PLWH changed a lot after that period. Furthermore, the outcome domains depression, anxiety and quality of life were specified a priori. Only the outcome psychological well-being was first intended for stress-related outcomes, but this was a rather small category, so we decided to enlarge it. Positive and negative affect, psychological functioning and general mental health were examples of concepts that were included in the outcome psychological well-being. Studies that had posttraumatic stress disorder (PTSD) as an outcome were not included in the meta-analysis. Regarding the seventh criterion, authors of the included papers were contacted to retrieve data that was not available in the paper. When the authors did not respond to the requests (even after reminder e-mails) and the data to calculate effect sizes was not available, the study was not included in the meta-analysis.

The first step was the selection of studies on title and abstract, and was performed by two persons (first and fourth author). The first 100 studies were selected by both authors independently, to determine the agreement among selectors, which was substantial [19], Cohen’s kappa = 0.80. Thereafter, both selected half of the remaining studies. For the second step of selecting studies, based on full text, the studies were divided among three persons (first and fourth author and a Master student in clinical psychology). The inclusion criteria, as described above, were used in the following order to ensure a fast and equal decision: 6, 3, 1, 4, 2, 5, 7. When there was doubt about including a study, the paper was discussed with one or more of the other authors to make a decision.

### Problems of Multiplicity

Some decisions had to be made when multiple papers were published about the same data or when multiple measuring instruments, time points or interventions were used in one study. When there were multiple papers about the same data, the paper with the most relevant outcome data was used as the main paper. Other papers were used to add information that was not present in the main paper. In addition, when there were multiple measuring instruments for one outcome, two instruments were included in the analysis and their data was averaged. The instruments that were most validated and comparable to other studies were chosen. When there were assessments at more than one time point after the termination of the intervention, the first time point (first post-test) was included in the main analysis. Moreover, we did investigate overall differences between time points post intervention. For this analysis, time points were classified into these categories: 0–3 months post intervention, more than 3–6 months post intervention, more than 6–9 months post intervention, and more than 9 months post intervention. When two assessments occurred in one time period, both were included in the analysis and the data was averaged. At last, when there were multiple intervention and/or control conditions in a study, they were all included in the analysis and coded as intervention or control conditions. In the analysis concerning the overall effectiveness of psychosocial interventions on mental health, the data of multiple intervention conditions was averaged. To investigate which interventions and techniques were most effective in the moderator analysis, all interventions were investigated separately. Therefore, some studies were represented multiple times in this analysis. To be included as an intervention condition, the intervention should have a psychosocial component. In the control condition, people were put on a waiting list, received standard care or were in an active control condition. This last category included for example (psycho)education, support and telephone check-ups.

### Data Extraction and Coding

We developed a protocol to extract the data from the articles. The following information was extracted from the papers: year of publication, baseline scores on outcome variables, post-treatment results, follow-up results, country of data collection, years of recruitment, study setting (inpatient; outpatient; combination), number of participants in each group, percentage attrition, percentage females, mean age, percentage MSM, percentage participants with AIDS, mean number of years with HIV, percentage participants that use ART, screening on depression (yes; no), intervention type (symptom-oriented intervention; supportive intervention; meditation intervention), intervention techniques (relaxation; CBT; motivational interviewing; stress-management), intervention duration (total duration in hours; duration in weeks; number of sessions; duration of one session), therapy provider [psychologist/psychotherapist; counsellor (e.g., nurse, HIV specialist, social worker, trained facilitator); peer; none (e.g., computer); other], intervention format [group; individual; combination; other (e.g., family interventions)], primary focus of intervention (mental health; no mental health), primary outcome (mental health; no mental health), theory content of intervention (theory-driven; not theory-driven), type of control group (waiting list; standard care; active control group), length of follow-up, type of analysis [intent-to-treat (ITT); no ITT], and study quality (see next paragraph).

The intervention type variable included three categories: symptom-oriented interventions, supportive interventions and meditation interventions. These categories were created post hoc, after examining the content of the included interventions. The category symptom-oriented interventions included mostly cognitive and/or behavioral therapy, stress-management, and interpersonal therapy. Furthermore, the category supportive interventions consisted of (peer) support and psycho-education, and the category meditation interventions included interventions that incorporated mindfulness, meditation, or relaxation. Since the symptom-oriented interventions used various psychological techniques, it was further investigated whether symptom-oriented interventions that used a specific technique would have larger effects than symptom-oriented interventions that did not use this specific technique. This was investigated in the symptom-oriented interventions only, because the supportive and meditation interventions mostly did not make use of additional psychological techniques. The assessed intervention techniques were relaxation, CBT (defined as containing cognitive and/or behavioral techniques), motivational interviewing and stress-management. One symptom-oriented intervention may use multiple psychological techniques. For example, a symptom-oriented intervention may include CBT techniques and relaxation techniques. The explanation of intervention types and intervention techniques is depicted in Table 1. The variable theory content of intervention contains two categories: (1) it was described that the intervention was based on theory or a theoretical model (e.g., social cognitive theory or the health belief model) or (2) it was not described that the intervention was based on theory or a theoretical model. The type of control group was categorized as waiting list, standard care or an active control condition. Standard care included for example standard medical care and referral to mental health services when needed. The last category included for instance (psycho)education, support and telephone check-ups.

**Table 1:** Intervention types and intervention techniques

Intervention types	Intervention techniques (in symptom-oriented interventions only)
Symptom-oriented intervention (e.g., cognitive and/or behavioral therapy, stress-management, interpersonal therapy)	Relaxation
Supportive intervention (e.g., support, psycho-education)	CBT
Meditation intervention (e.g., mindfulness, meditation, relaxation)	Motivational interviewing
	Stress-management

The following information was asked from the authors, when it was not available in the paper: baseline, post-treatment and follow-up data, years of recruitment, number of participants in each group, percentage attrition, percentage females, mean age, intervention duration, therapy provider, intervention format, type of control group, length of follow-up and type of analysis. The data was extracted by two persons (first author and a psychologist). Both coded a portion of the studies and 17 of the studies (27%) were coded by both authors. The intraclass correlation coefficient was calculated for the agreement on continuous variables, which was 0.99. For the categorical variables a Cohen’s kappa was calculated, this was 0.72, which is substantial.

### Study Quality

Study quality was assessed by using two methods: the Cochrane Collaboration’s tool for assessing risk of bias [20] and three criteria from a review about defining empirically supported psychological treatments [21]. The Cochrane Collaboration’s tool for assessing risk of bias consists of six domains. Four domains were used in this study: (1) sequence generation for allocation to conditions; (2) concealment of allocation to conditions; (3) addressing incomplete outcome data; and (4) selective outcome reporting. The domain blinding of participants and researchers was not used, because in almost all studies participants and researchers could not be blinded to the allocation to conditions. Furthermore, the domain other sources of bias was not used, because in most studies there were no other sources of bias. On each domain, a study received the judgement low risk of bias (+), high risk of bias (−) or unclear risk of bias (?) using the criteria from the tool.

Furthermore, we used three criteria from a review about defining empirically supported psychological treatments, to assess the quality of administering the intervention: (1) the availability of a treatment manual that was followed (published or designed for the study); (2) the use of a training for the therapy providers (for the study or general training); and (3) treatment integrity was checked during the study (e.g., supervision of therapy providers, recording of sessions, checking of protocol adherence). For each criterion a study received a judgement of yes (+, low risk of bias), no (−, high risk of bias), unclear (?) or not applicable (NA; e.g., when the intervention is a self-help program). Two persons (first author and a psychologist) rated the quality of the studies. Both rated a portion of the studies and 17 studies were rated by both authors to calculate their agreement. Cohen’s kappa was 0.67, which is substantial.

### Moderators

The following moderators were investigated: country of data collection, first year of participant recruitment, percentage attrition, percentage females, mean age, percentage MSM, percentage participants with AIDS, mean number of years with HIV, percentage participants that use ART, screening on depression, intervention techniques, intervention duration, therapy provider, intervention format, primary focus intervention, primary outcome mental health, theory content of intervention, type of control group, type of analysis and study quality.

For the moderator analyses with continuous variables, the assumptions for meta-regression were checked (normality and linearity). None of the variables met both assumptions. Therefore, the continuous variables were transformed into categorical variables. The categorization was based on statistical and content related reasons. The variable first year of recruitment was categorized into three periods: 1996–2001, 2002–2006 and 2007–2012. The variable percentage of drop-out was separated into three categories: 0–10, 10–20 and >20%, as was the variable percentage of females: 0–20%, 20–80 and 80–100%. The variable mean age was divided based on a median split: <42.40 and ≥42.40 years. The variable percentage MSM was divided into two categories: 0 and >0% (because most studies had no MSM, so the median was 0%). The variable percentage of people with AIDS was separated into two categories based on a median split: <40 and ≥40%, as was the variable number of years with HIV: <10.02 and ≥10.02 years and the variable percentage of people on ART: <87 and ≥87%. Finally, the variable total intervention duration was divided into four categories: 1–5, 5–12, 12–18 and 18–30 h.

Study quality was included as a moderator in the analysis. When a study met 0–2 out of seven quality criteria (0–2 times a +), the study was classified as a study with low quality. When a study met 3–4 quality criteria, the study was classified as a study with medium quality and when 5–7 criteria were met, the study was classified as a study with high quality. A rating of unclear risk of bias was scored as a high risk of bias (−) in this classification. For studies with a judgement of NA on the three criteria regarding the quality of administering the intervention, a low quality rating was given to studies which had a low risk of bias rating on 0–1 on the four other quality criteria, a medium quality rating was given to studies which had a low risk of bias on 2 of the other quality criteria and a high quality rating was for the studies which had a low risk of bias on 3–4 of the other quality criteria.

### Data Analysis

The program comprehensive meta-analysis (CMA; version 3) was used for the analysis. Hedges’ *g* was calculated as a measure of effect size. Baseline, post intervention and follow-up means, standard deviations, sample sizes and/or other available data were used to calculate effect sizes (e.g. *F*, *t* or *p* values). One study [22] reported median decreases in depression scores, instead of mean decreases. These medians were entered into CMA, because the means could not be retrieved. Also, five studies [23–27] found no differences between intervention and control conditions on one or more outcome measures, but no data was available. The effect sizes of these outcome measures of the studies were set at zero. Cohen’s guidelines were applied to interpret effect sizes: 0.2 may indicate a small effect size, 0.5 may indicate a medium effect size and 0.8 may indicate a large effect size [28]. Two-tailed *p*-values were used in all analyses. In CMA, a correlation between pre- and posttest should be indicated for each study. Since this correlation was rarely reported in study papers, this was set at 0.5 (as suggested by [29]). Standardized residuals were inspected to find outliers, defined as studies with standardized residuals larger than |3| [30].

A random effects model was used for the main analysis to estimate the pooled effect size of psychosocial interventions on mental health (expressed as Hedges’ *ĝ*). Separate analyses were conducted for each outcome (depression, anxiety, quality of life, and psychological well-being), intervention type (symptom-oriented intervention, supportive intervention, and meditation intervention) and time point (0–3 months post intervention, 3–6 months post intervention, 6–9 months post intervention, and >9 months post intervention). The random effects model was used because we assumed heterogeneity across studies. To investigate the presence and amount of heterogeneity, *Q* and *I* ^2^ were calculated. When *Q* is significant, this means that the results of the studies are probably not consistent. The amount of heterogeneity can be identified with *I* ^2^. Values of 25% indicate low heterogeneity, 50% indicates moderate heterogeneity and 75% indicates high heterogeneity [31].

For the moderator analysis, a mixed effects model was used, in which the random effects model was used to combine studies in one subgroup and a fixed effects model was used to compare across subgroups [32]. In CMA, the mixed and random effects option was set to: do not assume a common among-study variance component across subgroups (do not pool within-group estimates of tau-squared).

To examine the presence of publication bias different methods were used. First, a funnel plot was created, where the standard error is plotted as a function of effect size. Studies with small standard errors (large studies in general) are displayed at the top of the plot and studies with large standard errors (small studies in general) are displayed at the bottom of the plot. When the studies are symmetrically distributed around the pooled effect size estimate, there is no indication of publication bias. When it seems that studies are missing on the lower left side, this may be an indication of publication bias (small studies with results lower than the pooled estimate are missing). Second, Egger’s test of the intercept [33] was used to statistically test for publication bias. There is an indication of publication bias when the test is significant. Last, Duval and Tweedie’s trim and fill analysis [34] was used to investigate whether it was necessary to impute studies in the funnel plot due to publication bias. After the imputation of missing studies, an adjusted effect size was calculated.

## Results

**Fig. 1:**
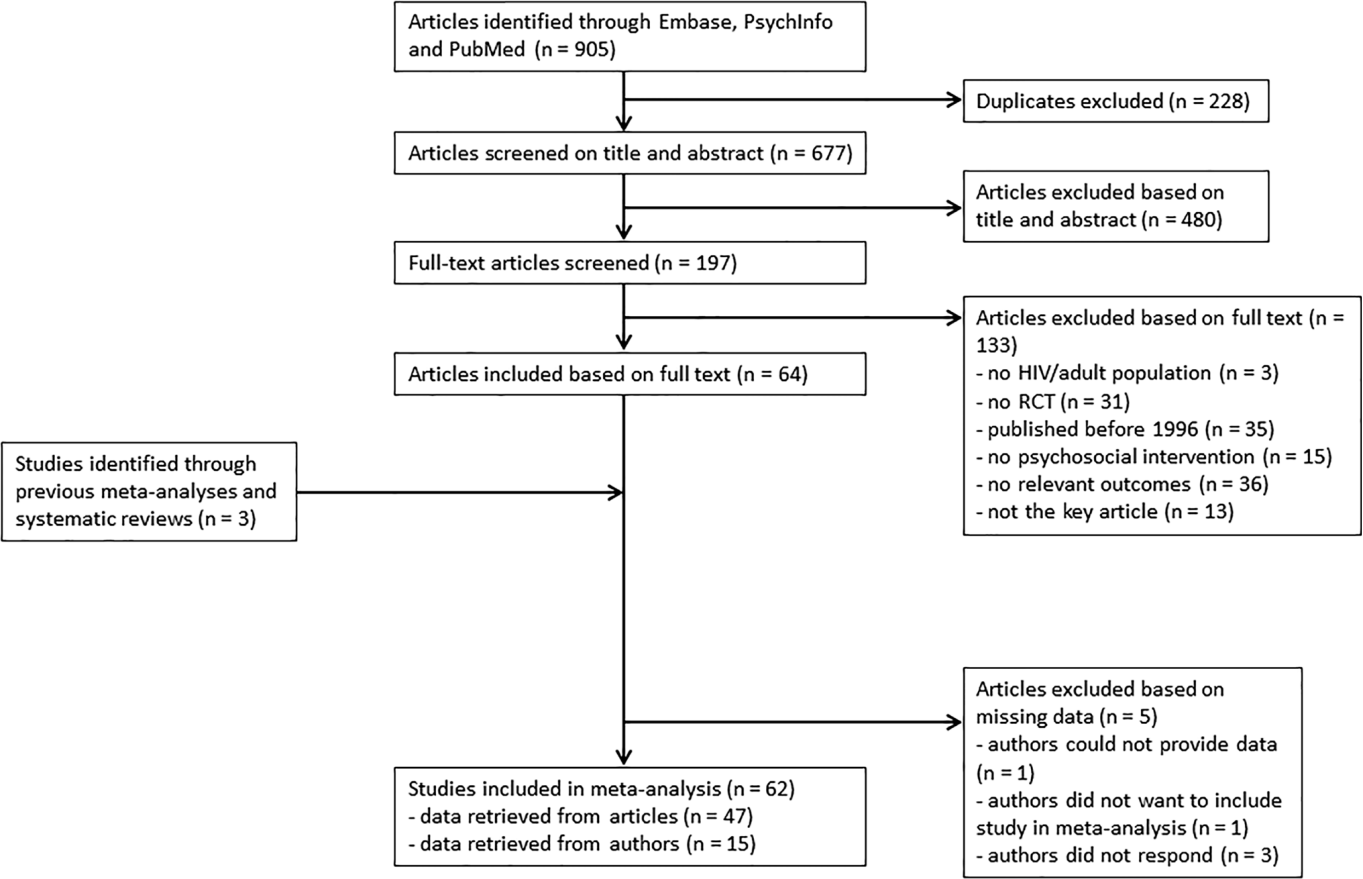
Flow chart of study inclusion and exclusion

Through electronic databases, 905 articles were identified (see flow-chart of study inclusion and exclusion in Fig. 1). After removal of duplicates (228), 677 articles were screened on title and abstract. Thereafter, 197 articles were screened on full text. After this screening, 64 studies met the inclusion criteria. In addition, three studies were found in previous meta-analyses and systematic reviews. From 20 of the 67 studies, data to calculate effect sizes was not present in the paper. Therefore, the authors were contacted to obtain these data. Of 15 studies, the authors were able to provide the data, one author could not provide the data, the authors of one study did not want to be included in the meta-analysis (because study aim did not fit with the aim of the meta-analysis), and three authors did not respond. In total, 62 studies were included in the meta-analysis.

### Study Characteristics

The characteristics of all included studies are presented in Table 2. In total, 10,307 participants were randomized to intervention and control conditions (range 12–936; *k* = 62). Drop-out (the percentage of participants that did not complete the first posttest) ranged from 0 to 55%, with a mean of 18% (*SD* = 11.93; *k* = 58). Seven studies (7/60) included only males and 13 included only females, the percentage of females in the included studies was 44% on average (*SD* = 34.54). The mean of the mean age of participants across studies was 42.01 years (*SD* = 5.98, range of the mean: 26.00–59.00 years, *k* = 54). The mean percentage of MSM in the studies that reported on it was 31% (*SD* = 38.27; *k* = 22). Across the studies that documented it, the mean percentage of people with AIDS was 45% (*SD* = 28.59; *k* = 15) and the mean duration of HIV was 9.81 years (*SD* = 3.59, range of the mean: 0–18 years; *k* = 28). Most participants in the studies that reported on it used ART, the mean percentage of people that used it is 76% (*SD* = 34.28; *k* = 28). For some characteristics, the number of studies that reported on it was very low. Therefore, these results should be interpreted with caution.

**Table 2:** Characteristics of the included studies

Authors and year of publication	Country and recruitment period^a^	*N* after randomization and sample description^b^	Mean age (*SD*)	Female (%)	Intervention: name (N)^c^, type (T)^d^ (techniques)^e^, provider (P)^f^, duration (D)^g^, setting (S)^h^	Control group	Outcomes and measures^i^	Follow-up	Drop-out (%)^j^	ITT^k^
Balfour et al., 2006 [35]	Canada 2000–2004	*N* = 27, PLWH diagnosed with depression	NR	NR	N: Supportive Therapy for Adherence; T: Supportive intervention; P: Psychologist; D: 5 h; S: Individual	Standard care	Depression: CES-D	–	0	Yes
Berger et al., 2008 [36]	Switzerland 2003–2004	*N* = 104, PLWH on ART	43.96	14	N: Cognitive behavioral stress management; T: Symptom-oriented intervention (CBT, relaxation, stress-management); P: Psychotherapist; D: 24 h; S: Group	Standard care	Depression: HADS	6 and 12 months	26	Yes
							Anxiety: HADS			
							Quality of life: MOS-HIV			
Boivin et al., 2013a [37]	Uganda 2008–2010	*N* = 71, mothers with HIV	33.69	100	N: Mediational intervention for sensitizing caregivers; T: Supportive intervention; P: Field trainer; D: 26 h; S: Individual	Active control group: information	Depression: HSCL-25	–	17	NR
							Anxiety: HSCL-25			
Boivin et al., 2013b [38]	Uganda 2008–2010	*N* = 109, mothers with HIV	NR	100	N: Mediational intervention for sensitizing caregivers; T: Supportive intervention; P: Field trainer; D: 26 h; S: Individual	Active control group: information	Depression: HSCL-25	–	0	Yes
							Anxiety: HSCL-25			
Bormann et al., 2006 [39]	USA 2003–2004	*N* = 93, PLWH for more than 6 months	42.9 (6.84)	81	N: Spirtual mantram repetition; T: Meditation; P: Nurse; D: 10 h; S: Group and individual	Active control group: information and discussion	Depression: CES-D	3 months	20	Yes
							Anxiety: STAI			
							Quality of life: Q-LES-Q			
							Well-being: PSS, FACIT-SpEx			
Brazier et al., 2006 [23]	Canada 2000	*N* = 62, PLWH	NR	NR	N: The art of living with HIV program; T: Meditation; P: NR; D: NR; S: Group	Standard care	Quality of life: MOS-HIV	–	24	NR
							Well-being: MHI, DSI			
Brown et al., 2011 [40]	USA 2009	*N* = 60, females with HIV	44.7 (8.80)	100	N: Computerized stress management training; T: Symptom-oriented intervention (CBT, relaxation, motivational interviewing, stress-management); P: Computer; D: 2 h; S: Individual	Waiting list	Depression: CES-D, BSI	–	2	NR
							Anxiety: BSI, POMS			
							Well-being: PSS, HIV-related life- stressor burden questionnaire			
Carrico et al., 2006 [41]	USA 1998–2004	*N* = 130, gay/bisexual men with HIV	41.6 (8.60)	0	N: Cognitive behavioral stress management; T: Symptom-oriented intervention (CBT, relaxation, stress-management); P: Psychologist; D: 22.5 h; S: Group	Active control group: information	Depression: POMS	–	25	Yes
Carrico et al., 2009 [42]	USA 2000–2002	*N* = 936, PLWH that had unprotected sex	39.8	21	N: Healthy living project: CBT; T: Symptom-oriented intervention (CBT); P: NR; D: 22.5 h; S: Individual	Waiting list	Depression: BDI	7.5 and 12.5 months	20	Yes
							Anxiety: STAI			
							Quality of life: SF-36			
							Well-being: PSS			
Chan et al., 2005 [43]	China NR	*N* = 16, males with HIV	38.15 (8.03)	0	N: Group CBT; T: Symptom-oriented intervention (CBT, relaxation, stress-management); P: Psychologist; D: 14 h; S: Group	Waiting list	Depression: CES-D	–	19	No
							Quality of life: SF-36			
Chang et al., 2007 [44]	USA 2003–2004	*N* = 119, PLWH that suffer from HIV-related symptoms	45.5 (7.5)	15	N: Relaxation during acupuncture treatment; T: Meditation; P: Tape; D: 10.5 h; S: Individual	Standard care	Quality of life: MOS-HIV, FAHI	–	33	NR
Chhatre et al., 2013 [45]	USA 2011	*N* = 22, PLWH on ART	49.9 (5.7)	18	N: Transcendental meditation; T: Meditation; P: Certified instructor; D: 28 h; S: Group	Active control group: information	Depression: CES-D	–	9	No
							Quality of life: SF-36, FAHI			
							Well-being: PSS			
Côté & Pepler, 2002 [46]	Canada 1996–1998	*N* = 90, males with HIV	40	0	N1: Cognitive coping skills intervention; T1: Symptom-oriented intervention (CBT); N2: Expression of emotions intervention; T2: Supportive intervention; P: Nurse; D: 1.25 h; S: Individual	Waiting list	Well-being: PANAS	–	NR	No
Duncan et al., 2012 [47]	USA 2006–2008	*N* = 76, PLWH with distress	48.06 (7.93)	16	N: Mindfulness based stress reduction; T: Meditation; P: Mindfulness teacher; D: 30 h; S: Group & individual	Standard care	Depression: BDI	3 months	14	Yes
							Well-being: PSS, PANAS			
Eller et al., 2013 [48]	South Africa, Puerto Rico, USA 2005–2007	*N* = 222, PLWH with depression	43.15 (9.59)	42	N: HIV/AIDS symptom management manual; T: Supportive intervention; P: Self-help; D: NR; S: Individual	Active control group: information	Depression: depressive symptom intensity and frequency	2 months	18	NR
Erlen et al., 2001 [49]	USA 1998	*N* = 20, people with AIDS	42.05	20	N: Life review; T: Symptom-oriented intervention; P: Nurse; D: 6 h; S: Individual	Standard care	Depression: CES-D	3 and 12 months	NR	NR
							Quality of life: Ferrans and Powers Quality of Life Index			
Fife et al., 2008 [50]	USA NR	*N* = 80, PLWH	NR	30	N: A psychosocial education model; T: Symptom-oriented intervention (stress-management); P: NR; D: 8 h; S: Other (with partner)	Active control group: telephone support	Depression: PANAS	3 months	33	No
							Anxiety: PANAS			
							Well-being: PANAS			
Gayner et al., 2012 [51]	Canada 2004–2007	*N* = 117, males with HIV	43.79 (7.08)	0	N: Mindfulness based stress reduction; T: Meditation; P: Social worker & psychologist; D: 30 h; S: Group	Standard care	Depression: HADS	8 months	12	Yes
							Anxiety: HADS			
							Well-being: PANAS			
Heckman & Carlson, 2007 [52]	USA 1999–2002	*N* = 299, PLWH	43.10	30	N1: Telephone coping improvement group; T1: Symptom-oriented intervention (CBT); N2: Telephone information support group; T2: Supportive intervention; P: Practitioner; D: 12 h; S: Group	Standard care	Depression: BDI	4 and 8 months	14	Yes
							Quality of life: FAHI			
							Well-being: SCL-90, HIV-related life stressor burden scale			
Heckman et al., 2011 [53]	USA 2004–2007	*N* = 295, PLWH with depressive symptoms, age > 49 years	55.30 (4.80)	33	N1: Coping improvement group; T1: Symptom-oriented intervention (stress-management); N2: Interpersonal support group; T2: Supportive intervention; P: Social worker & psychologist; D: 18 h; S: Group	Active control group: telephone check-ups and individual therapy when needed	Depression: GDS	4 and 8 months	17	Yes
Heckman et al., 2013 [54]	USA 2008–2010	*N* = 361, PLWH with depressive symptoms, age > 49 years	59.00 (5.10)	39	N1: Telephone coping effectiveness training; T1: Symptom-oriented intervention (CBT, stress-management); N2: Telephone supportive-expressive group; T2: Supportive intervention; P: Therapist; D: 18 h; S: Group	Standard care	Depression: GDS	4 and 8 months	8	Yes
Hersch et al., 2013 [55]	USA 2010–2011	*N* = 168, PLWH on ART	46.34 (9.84)	24	N: Life steps intervention; T: Symptom-oriented intervention (CBT, relaxation, stress-management); P: Computer; D: NR; S: Individual	Waiting list	Well-being: HIV/AIDS stress scale, PANAS	3 and 6 months	9	Yes
Ironson et al., 2013 [56]	USA 2004–2009	*N* = 244, PLWH in mid-range of illness	42.80 (8.80)	39	N: Augmented trauma writing; T: Symptom-oriented intervention; P: Self-help; D: 2 h; S: Individual	Active control group: daily event writing	Depression: HAM-D	6 and 12 months	12	Yes
Jensen et al., 2013 [57]	USA 2000–2004	*N* = 72, females with HIV	31.27 (8.41)	100	N: Cognitive behavioral stress management; T: Symptom-oriented intervention (CBT, relaxation, stress-management); P: Psychologist; D: 22.5 h; S: Group	Active control group: psycho-education	Depression: BDI	6 months	NR	Yes
							Well-being: FACIT-SpEx			
Kaaya et al., 2013 [58]	Tanzania 2001–2004	*N* = 331, pregnant women with HIV	26.00	100	N: Counselling; T: Supportive intervention; P: Social worker/nurse; D: NR; S: Group	Standard care	Depression: HSCL-25	–	43	No
Kalichman et al., 2005 [59]	USA 1999–2000	*N* = 125, PLWH that had sex with nonconcordant sex partners	NR	30	N: Healthy relationships intervention; T: Symptom-oriented intervention (CBT, stress-management); P: NR; D: 10 h; S: Group	Active control group: information and support	Depression: BDI	3 and 6 months	35	No
							Anxiety: BSI			
							Well-being: HIV-related stress			
Klein et al., 2013 [60]	USA 2011	*N* = 175, African American females with HIV	40.70 (8.50)	100	N: Women involved in life learning from other women; T: Symptom-oriented intervention (relaxation, stress-management); P: Computer; D: 2 h; S: Individual	Active control group: information and discussion	Well-being: Willow Stress Scale	–	4	Yes
Kraaij et al., 2010 [61]	The Nether-lands 2008	*N* = 73, PLWH	49.48 (8.15)	11	N1: CBT self-help; T1: Symptom-oriented intervention (CBT, relaxation); N2: Computerized structured writing; T2: Symptom-oriented intervention; P: Self-help; D1: 16 h; D2: 2 h; S: Individual	Waiting list	Depression: HADS	–	25	No
Lechner et al., 2003 [62]	USA NR	*N* = 330, women with AIDS	39.60 (7.14)	100	N: Cognitive behavioral stress management + expressive/supportive therapy; T: Symptom-oriented intervention (CBT, relaxation, stress-management); P: Psychologist; D: 20 h; S: Individual	Active control group: psycho-education	Quality of life: MOS-HIV	–	18	Yes
Li et al., 2010 [63]	Thailand 2007	*N* = 507, PLWH	37.40 (6.60)	67	N: Behavioral intervention; T: Symptom-oriented intervention (stress-management); P: Nurse/counsellor; D: 18 h; S: Group	Standard care	Quality of life: MOS-HIV	6 months	2	NR
Lovejoy, 2012 [64]	USA 2009–2010	*N* = 100, PLWH that had unprotected sex, age > 44 years	53.80 (4.90)	46	N1: Motivational Interviewing (1 session); T1: Symptom-oriented intervention (motivational interviewing); N2: Motivational Interviewing (4 sessions); T2: Symptom-oriented intervention (motivational interviewing); P: Psychologist; D1: 0.81 h; D2: 2.72 h; S: Group	Active control group: encouraged to obtain information and support	Depression: DASS	3 months	8	Yes
							Anxiety: DASS			
							Well-being: DASS			
McCain et al., 2008 [65]	USA 2000–2004	*N* = 387, PLWH	42.20	40	N1: Cognitive behavioral relaxation; T1: Symptom-oriented intervention (CBT, relaxation, stress-management); N2: Tai Chi Training; T2: Meditation; N3: Spirtual growth; T3: Meditation; P: Investigator; D: 15 h; S: Group	Waiting list	Quality of life: FAHI	6 months	NR	Yes
Miles et al., 2003 [66]	USA 1997–2000	*N* = 109, African America females with HIV	35.50 (8.49)	100	N: Maternal self-care symptom management; T: Supportive intervention; P: Nurse; D: NR; S: Individual	Standard care	Depression: CES-D, POMS	5 months	32	Yes
							Anxiety: POMS, HIV worry scale			
							Quality of life: MOS-HIV			
Miller et al., 2005 [67]	USA 1999–2000	*N* = 12, PLWH	39.42 (9.75)	25	N: Supportive-affective group experience; T: Supportive intervention; P: Mediators; D: 18 h; S: Group	Active control group: sent self-help materials	Depression: BDI	–	25	No
							Anxiety: STAI state			
							Well-being: SWS; DDS			
Mitrani et al., 2012 [68]	USA 2003–2007	*N* = 126, females with HIV and substance use diagnosis	42.60 (7.50)	100	N: Structural Ecosystems Therapy; T: Symptom-oriented intervention (CBT); P: Social worker; D: 5.2 h; S: Other (family)	Active control group: information	Depression: BSI, SIGH-AD	4 and 8 months	8	Yes
							Anxiety: BSI, SIGH-AD			
							Well-being: PSS			
Murphy et al., 2002 [69]	USA 1999–2000	*N* = 52, PLWH with adherence problems	39.00 (6.88)	12	N: CBT for adherence; T: Symptom-oriented intervention (CBT); P: NR; D: NR; S: Group & individual	Standard care	Depression: CES-D, RAND mental health inventory	3 months	37	No
							Anxiety: health-related anxiety, RAND mental health inventory			
Murphy et al., 2011 [70]	USA 2007–2009	*N* = 80, mothers with HIV	37.40 (6.80)	100	N: Teaching, Raising and Communicating with Kids program; T: Supportive intervention; P: Social worker; D: 3.25 h; S: Group	Standard care	Depression: RAND mental health inventory	5 and 8 months	14	Yes
							Anxiety: RAND mental health inventory			
							Quality of life: SF-36			
O’Leary et al., 2005 [71]	USA 2000–2001	*N* = 811, males with HIV that had sex with male partners whose HIV status was seronegative or unknown	41.00 (7.90)	0	N: Peer-led behavioral intervention; T: Supportive intervention; P: Peer; D: 18 h; S: Group	Active control group: information and discussion	Depression: BSI	6 months	15	No
							Anxiety: BSI			
Olley, 2006 [72]	Nigeria NR	*N* = 67, PLWH	27.40 (8.10)	56	N: Psycho-education; T: Supportive intervention; P: NR; D: 4 h; S: Individual	Active control group: discussion and support	Depression: BDI	1 month	7	NR
							Anxiety: CCEI			
Pacella et al., 2012 [73]	USA 2005–2008	*N* = 66, PLWH with PTSD and on ART	46.37 (6.30)	37	N: Prolonged exposure; T: Symptom-oriented intervention (CBT); P: Psychologist; D: 17.5 h; S: Individual	Active control group: telephone check-ups	Depression: CES-D	3 months	29	Yes
							Well-being: PTCI			
Peltzer et al., 2012 [74]	South Africa 2010	*N* = 152, PLWH with adherence problems	36.90 (6.50)	65	N: Medication adherence intervention; T: Supportive intervention; P: Counsellor; D: 3 h; S: Group	Standard care	Depression: BDI	3 months	3	NR
Petersen et al., 2014 [75]	South Africa 2012–2013	*N* = 76, PLWH diagnosed with depression	37.59 (10.36)	74	N: Group-based interpersonal psychotherapy; T: Symptom-oriented intervention (CBT); P: HIV specialist; D: 8 h; S: Group	Standard care	Depression: PHQ-9	–	55	No
							Anxiety: HSCL-25			
Ransom et al., 2008 [76]	USA 2006–2007	*N* = 79, PLWH with depression	44.40 (8.60)	16	N: Telephone interpersonal psychotherapy; T: Symptom-oriented intervention; P: Psychologist; D: 5 h; S: Individual	Standard care	Depression: BDI	–	16	Yes
							Well-being: OQ			
Rao et al., 2009 [24]	USA 2006–2007	*N* = 79, PLWH	42.00 (10.00)	25	N: Art therapy; T: Symptom-oriented intervention; P: Art therapist; D: 1 h; S: Individual	Active control group: videotape	Anxiety: STAI state	–	4	No
Rotherham-Borus et al., 2012 [25]	USA 2005–2006	*N* = 339, mothers with HIV	40.20 (8.20)	100	N: Family CBT; T: Symptom-oriented intervention (CBT); P: NR; D: 28 h; S: Other (group and family)	Waiting list	Well-being: BSI	6 and 12 months	8	Yes
Safren et al., 2009 [77]	USA 2002–2004	*N* = 45, PLWH with depression and on ART	NR	16	N: CBT for adherence and depression; T: Symptom-oriented intervention (CBT, relaxation, motivational interviewing); P: Psychologist; D: 8.68 h; S: Individual	Active control group: single session about adherence	Depression: BDI, HAM-D	3 and 9 months	7	Yes
							Well-being: CGI			
Safren et al., 2012 [78]	USA 2005–2008	*N* = 89, PLWH with depression and substance use disorder	46.85 (7.15)	39	N: CBT for adherence and depression; T: Symptom-oriented intervention (CBT, relaxation, motivational interviewing); P: Psychologist; D: 6.64 h; S: Individual	Active control group: single session about adherence	Depression: BDI, MADRS	3 and 9 months	9	Yes
							Well-being: CGI			
Sarna et al., 2008 [22]	Kenya 2003–2004	*N* = 234, PLWH that started with ART	37.15 (7.90)	64	N: Adherence intervention; T: Supportive intervention; P: Nurse; D: NR; S: Individual	Active control group: counselling	Depression: BDI	5.5 and 11 months	15	NR
SeyedAlinaghi et al., 2012 [79]	Iran 2008–2010	*N* = 245, PLWH	35.10 (6.50)	31	N: Mindfulness based stress reduction; T: Meditation; P: Psychologist; D: 25.75 h; S: Group	Active control group: information and support	Depression: SCL-90	3, 6, 9 and 12 months	29	No
							Anxiety: SCL-90			
Shuter et al., 2014 [80]	USA 2012–2013	*N* = 138, PLWH interested in quitting smoking	45.62 (9.91)	43	N: Positively smoke free on the web; T: Symptom-oriented intervention; P: Computer; D: 2 h; S: Individual	Active control group: advice and brochure	Depression: CES-D	2.5 months	9	Yes
							Anxiety: GAD-7			
							Well-being: PSS			
Sikkema et al., 2004 [81–84]	USA 1997–1999	*N* = 267, PLWH that lost a loved one to AIDS	40.10 (7.02)	35	N: CBT; T: Symptom-oriented intervention (CBT, stress-management); P: Therapist; D: 18 h; S: Group	Standard care	Depression: SCL-90, HAM-D	4, 8 and 12 months	12	No
							Anxiety: SCL-90, HAM-A			
							Quality of life: FAHI			
Simoni et al., 2007 [85]	USA 2000–2002	*N* = 136, PLWH on ART	42.60 (8.90)	45	N: Peer support; T: Supportive intervention; P: Peer; D: 12 h; S: Group & individual	Standard care	Depression: CES-D	3 months	23	No
Simoni et al., 2013 [86]	USA/Mexico 2009–2011	*N* = 40, PLWH with depressive symptoms and adherence problems	46.00 (10.60)	28	N: CBT for adherence and depression and electronic pillbox; T: Symptom-oriented intervention (CBT, relaxation, motivational interviewing); P: Psychologist; D: 9 h; S: Individual	Standard care	Depression: BDI, MADRS	3 months	15	Yes
Stein et al., 2007 [26]	USA 2001–2004	*N* = 177, PLWH with depressive symptoms	40.30 (7.40)	44	N: Telephone family intervention; T: Supportive intervention; P: Social worker, psychologist, nurse; D: 2.6 h; S: Other (with partner)	Waiting list	Depression: BDI	–	10	No
Szapocznik et al., 2004 [87]	USA 1996–1999	*N* = 209, African American mothers	36.00 (8.00)	100	N1: Structural Ecosystems Therapy; T1: Symptom-oriented intervention (CBT); N2: Attention condition; T2: Supportive intervention; P: Counsellor, social worker, therapist; D1: 12.45 h; D2: 5.74 h; S1: Other (family); S2: Individual	Standard care	Depression: BSI	9 months	8	Yes
							Anxiety: BSI			
Van Tam et al., 2012 [88]	Vietnam 2008–2009	*N* = 275, PLWH that started with ART	NR	32	N: Peer support for adherence; T: Supportive intervention; P: Peer; D: NR; S: Individual	Active control group: adherence counselling	Quality of life: WHOQOL-HIVBREF	–	17	No
Vidrine et al., 2006 [89]	USA 2004	*N* = 95, PLWH interested in quitting smoking	42.80 (8.10)	22	N: Telephone counselling for smoking cessation; T: Symptom-oriented intervention (CBT); P: Counsellor; D: NR; S: Individual	Active control group: advice and self-help	Depression: CES-D	–	16	NR
							Anxiety: STAI state			
Webel, 2010 [90]	USA 2008	*N* = 89, females with HIV	47.00 (8.16)	100	N: HIV symptom management; T: Symptom-oriented intervention (CBT, relaxation); P: Peer; D: 14 h; S: Group	Active control group: self-help manual	Quality of life: HIV/AIDS targeted quality of life instrument	1.75 months	48	Yes
Weber et al., 2004 [27]	Switzerland NR	*N* = 60, PLWH on ART	NR	17	N: CBT; T: Symptom-oriented intervention (CBT); P: Psychotherapist; D: NR; S: Individual	Standard care	Well-being: SCL-90	–	12	NR
Williams et al., 2005 [91]	USA 2001–2003	*N* = 58, people with advanced AIDS	45.09 (2.22)	21	N1: Meditation; T1: Meditation; N2: Meditation and massage; T2: Meditation; P1: Meditation teacher; P2: Meditation teacher and massage therapist; D1: 8.5 h; D2: 18.5 h; S: Group & individual	C1: active control group: provision of mental health services; C2: active control group: massage	Quality of life: MVQOLI	17 months	29	Yes
Williams et al., 2008 [92]	USA 2003–2006	*N* = 164, males with HIV and experience of childhood sexual abuse	43.50 (8.00)	0	N: Sexual Health Intervention for Men; T: Symptom-oriented intervention (CBT); P: Trained male facilitator; D: 12 h; S: Group	Active control group: information and discussion	Depression: CES-D	3 and 6 months	16	No
Williams et al., 2013 [93]	USA 2007–2011	*N* = 117, African American males with HIV that had unprotected sex	46.60 (8.30)	0	N: Enhanced sexual health intervention for men; T: Symptom-oriented intervention (CBT, stress-management); P: Trained male facilitator; D: 12 h; S: Group	Active control group: information	Depression: BDI	6 months	27	No

^a^ *NR* not reported in paper

^b^ N after randomization and sample description. With *ART* antiretroviral therapy, *PLWH* people living with HIV, *PTSD* posttraumatic stress disorder

^c^ *N* name of the intervention. With *CBT* cognitive behavioral therapy

^d^ *T* type of intervention

^e^Intervention techniques in symptom-oriented interventions. With *CBT* cognitive behavioral techniques

^f^ *P* provider intervention

^g^ *D* duration intervention

^h^ *S* setting intervention

^i^Outcomes and measures. With *BDI* Beck Depression Inventory, *BSI* Brief Symptom Inventory, *CCEI* Crown Crisp Experiential Index, *CES-D* Center for Epidemiologic Studies Depression scale, *CGI* Clinical Global Impression, *DASS* Depression Anxiety Stress Scales, *DDS* Death Distress Scale, *DSI* Daily Stress Inventory, *FAHI* Functional Assessment of HIV infection, *FACIT-SpEx* Functional Assessment of Chronic Illness Therapy Spiritual Well-being-Expanded, *GAD-7* Generalized Anxiety Disorder 7, *GDS* Geriatric Depression Scale, *HADS* Hospital Anxiety and Depression Scale, *HAM-A* Hamilton Anxiety Rating Scale, *HAM-D* Hamilton Depression Scale, *HSCL-25* Hopkins Symptom Checklist, *MADRS* Montgomery-Asberg Depression Rating Scale, *MHI* Mental Health Index, *MOS-HIV* Medical Outcomes Study HIV Health Survey, *MVQOLI* Missoula-VITAS Quality of Life Index, *OQ* Outcomes Questionnaire, *PANAS* Positive and Negative Affect Schedule, *PHQ-9* Patient Health Questionnaire 9, *POMS* Profile of Mood States *PSS* Perceived Stress Scale, *PTCI* Posttraumatic Cognitions Inventory, *SCL-90* Symptom Checklist 90, *SF-36* Short Form Health Survey, *SIGH-AD* Structured Interview Guide for the Hamilton Anxiety and Depression Subscales, *STAI* Spielberger State-Trait Anxiety Inventory, *SWS* Spiritual Well-being Scale, *Q-LES-Q* Quality of Life Enjoyment and Satisfaction Questionnaire

^j^Drop-out refers to the percentage of participants that did not complete the first posttest

^k^ *ITT* Intent-To-Treat analysis

The majority of studies was conducted in the USA and Canada (*k* = 46). Other countries of data collection were China, Iran, Kenya, Nigeria, South Africa, Switzerland, Tanzania, Thailand, The Netherlands, Uganda, and Vietnam. One study recruited participants in the USA and Mexico and one study in South Africa, Puerto Rico, and the USA. The years of participant recruitment ranged from 1996 to 2013 (*k* = 57). The majority of studies was conducted in an outpatient setting (58/62), only two studies were conducted in an inpatient setting and two studies combined inpatients and outpatients. Ten studies (10/62) incorporated the presence of depressive symptoms as an inclusion criterion. In the majority of studies (54/62), mental health (i.e., depression, anxiety, quality of life, or psychological well-being) was a primary outcome measure. Depression was measured in 47 studies, anxiety in 22, quality of life in 19, and psychological well-being in 25 studies. Some studies (31/62) used an active control condition, 9 studies used a waiting list condition, and 22 studies had a standard care control condition. Furthermore, more than half of the studies (32/51) used an ITT analysis, and included one or more follow-up assessments (40/62); 22 studies had one follow-up, 16 studies had two follow-ups, one study had 3 follow-ups, and one study had 4 follow-ups. The timing of follow-ups ranged from 1 to 17 months after treatment completion.

### Intervention Characteristics

The description of intervention characteristics was based on all interventions, so eight studies [46, 52–54, 61, 64, 87, 91] were represented twice in this analysis and one study [65] was represented three times, because multiple interventions were investigated in these studies. The letter m will be used to indicate the number of interventions. Regarding intervention types (see Table 1), a majority of the interventions were symptom-oriented (41/72), the rest were supportive (20/72), or meditation interventions (11/72). Regarding techniques used in symptom-oriented interventions (*m* = 41), CBT techniques were used in 29 interventions, relaxation techniques in 14, stress-management techniques in 16, and finally motivational interviewing techniques in 6 interventions. Almost two-thirds of the interventions (47/72) were focused on one of our outcome measures (depression, anxiety, quality of life, or psychological well-being). Studies that investigated interventions that were not focused on one of our outcome measures, were often aimed at medication adherence or sexual risk behavior, and sometimes at coping, disclosure, general health, family functioning, posttraumatic stress disorder symptoms, or smoking. A majority (44/72) of the interventions were theory-driven and 28 interventions were not theory-driven. Concerning the duration of the interventions, the total length ranged from 1 to 30 h (*m* = 62), with a mean of 12.63 (*SD* = 8.46). The duration of the intervention in weeks ranged from 1 to 54 (*M* = 12.20, *SD* = 13.27, *m* = 66) and the number of sessions ranged from 1 to 48 (*M* = 9.92, *SD* = 8.62, *m* = 67). The average length of one session was 1.37 h (*SD* = 0.66, range 15 min to 3 h, *m* = 63). Providers of the interventions were psychologists/psychotherapists (18/65), counsellors (e.g., nurses, 29/65), peers (4/65), none (e.g., computer interventions, 9/65), and other (e.g., investigators, 5/65). The format of the intervention was either individual (31/72), group (30/72), a combination of individual and group (6/72), or other (e.g., family interventions, 5/72).

### Quality of the Included Studies

The quality ratings of the studies are presented in Table 3. The first quality criterion, regarding the sequence generation for allocation to conditions, was reported in more than half of the studies (32/62), in the other studies it was unclear. The criterion about the concealment of allocation to conditions was often not reported, only 13 studies mentioned it, in the other studies it was unclear. Incomplete outcome data (the third criterion) was adequately addressed in half of the studies (31/62, e.g., with an ITT analysis), in 20 studies it was not adequately addressed and in 11 studies this was not clear. For most studies (58/62) there was no study protocol available, so the criterion of selective outcome reporting was unclear, only four studies had a rating of low risk of bias on this criterion.

**Table 3:** Quality of the included studies

Study	Sequence generation	Allocation concealment	Incomplete outcome data	Selective outcome reporting	Availability of treatment manual	Use of training for therapy providers	Treatment integrity was checked	Quality classification^a^
Balfour et al. [35]	+	+	+	?	+	+	?	High
Berger et al. [36]	+	+	+	+	+	+	?	High
Boivin et al. [37]	?	?	?	?	?	+	+	Low
Boivin et al. [38]	?	?	+	?	?	+	+	Medium
Bormann et al. [39]	+	?	+	?	+	?	+	Medium
Brazier et al. [23]	+	?	–	?	?	?	?	Low
Brown et al. [40]	+	?	+	?	NA	NA	NA	Medium
Carrico et al. [41]	+	?	+	?	+	?	+	Medium
Carrico et al. [42]	+	?	+	?	+	?	+	Medium
Chan et al. [43]	?	?	–	?	+	?	?	Low
Chang et al. [44]	+	+	+	?	NA	NA	NA	High
Chhatre et al. [45]	?	?	–	?	?	+	?	Low
Côté and Pepler [46]	?	?	–	?	+	?	+	Low
Duncan et al. [47]	+	?	?	?	?	+	?	Low
Eller et al. [48]	?	?	?	?	NA	NA	NA	Low
Erlen et al. [49]	?	?	?	?	?	+	+	Low
Fife et al. [50]	?	?	–	?	+	?	+	Low
Gayner et al. [51]	+	+	+	?	+	?	?	Medium
Heckman and Carlson [52]	?	?	+	?	+	?	+	Medium
Heckman et al. [53]	+	?	+	?	+	?	+	Medium
Heckman et al. [54]	+	?	+	?	+	–	+	Medium
Hersch et al. [55]	?	?	+	?	NA	NA	NA	Low
Ironson et al. [56]	?	?	+	?	NA	NA	NA	Low
Jensen et al. [57]	?	?	+	?	+	+	+	Medium
Kaaya et al. [58]	+	+	–	?	?	+	?	Medium
Kalichman et al. [59]	+	+	–	?	+	?	+	Medium
Klein et al. [60]	+	+	?	?	NA	NA	NA	Medium
Kraaij et al. [61]	+	?	–	?	NA	NA	NA	Low
Lechner et al. [62]	?	?	–	?	+	+	+	Medium
Li et al. [63]	?	?	?	?	+	+	?	Low
Lovejoy [64]	+	+	+	?	+	+	+	High
McCain et al. [65]	+	+	+	?	+	+	?	High
Miles et al. [66]	+	?	+	?	?	+	+	Medium
Miller et al. [67]	?	?	–	?	?	+	?	Low
Mitrani et al. [68]	?	?	+	?	+	+	+	Medium
Murphy et al. [69]	+	?	–	?	+	?	?	Low
Murphy et al. [70]	?	?	+	?	+	?	?	Low
O’Leary et al. [71]	+	?	–	?	+	+	?	Medium
Olley [72]	?	?	?	?	+	?	?	Low
Pacella et al. [73]	?	?	+	?	+	+	+	Medium
Peltzer et al. [74]	+	?	?	?	+	?	?	Low
Petersen et al. [75]	+	?	–	?	+	+	+	Medium
Ransom et al. [76]	?	?	+	?	+	+	+	Medium
Rao et al. [24]	?	?	+	?	?	?	?	Low
Rotherham-Borus et al. [25]	?	?	+	+	+	+	+	High
Safren et al. [77]	?	?	+	?	+	?	+	Medium
Safren et al. [78]	?	?	+	?	+	+	+	Medium
Sarna et al. [22]	+	+	?	?	?	+	+	Medium
SeyedAlinaghi et al. [79]	+	?	–	+	+	+	?	Medium
Shuter et al. [80]	+	?	+	+	NA	NA	NA	High
Sikkema et al. [81–84]	?	?	–	?	+	+	+	Medium
Simoni et al. [85]	+	+	–	?	?	+	+	Medium
Simoni et al. [86]	+	+	+	?	+	+	+	High
Stein et al. [26]	?	?	–	?	+	+	+	Medium
Szapocznik et al. [87]	+	?	+	?	+	+	+	High
Van Tam et al. [88]	?	?	–	?	?	+	+	Low
Vidrine et al. [89]	+	?	?	?	?	+	?	Low
Webel [90]	?	?	+	?	?	+	?	Low
Weber et al. [27]	+	+	?	?	+	+	+	High
Williams et al. [91]	+	?	+	?	?	+	?	Medium
Williams et al. [92]	?	?	–	?	?	+	?	Low
Williams et al. [93]	?	?	–	?	?	+	?	Low

+ low risk of bias, − high risk of bias; ? unclear risk of bias; NA = not applicable (e.g., when the intervention is a self-help program)

^a^quality classification, this was calculated by adding up the low risk of bias ratings (see “Method” section)

**Fig. 2:**
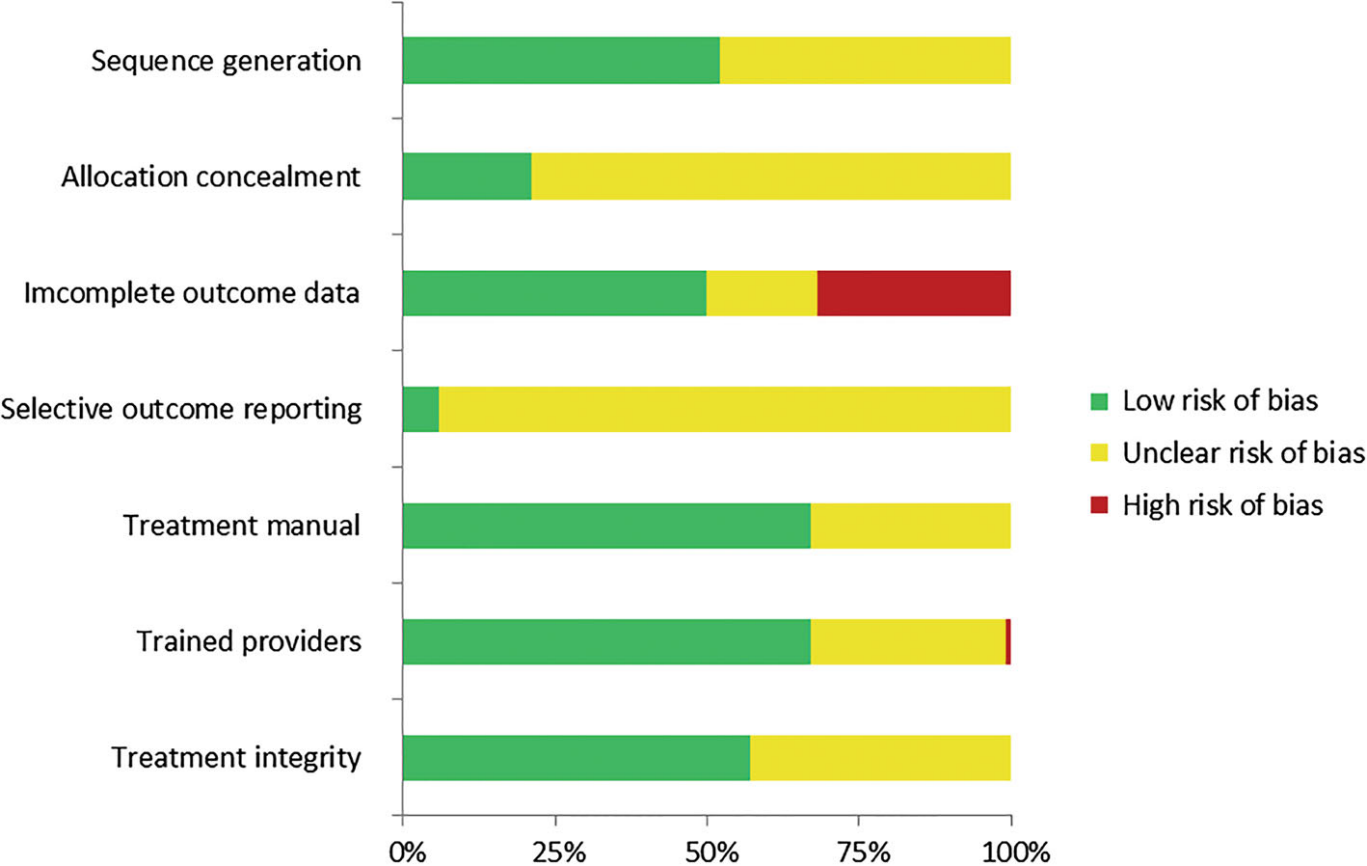
Risk of bias graph

Eight studies investigated an intervention without a provider (e.g., computer or self-help interventions). These studies were not coded on the quality of the administered intervention. In 36 of the 54 studies a treatment manual was available and followed and in 18 studies this was not described. Trained providers were used in 36 of the 54 studies, in 17 studies this was unclear and one study explained that they did not make use of trained providers. Finally, in 31 of the 54 studies treatment integrity was checked and in 23 studies this was not described. A summary of the ratings on all quality criteria is presented in Fig. 2. Regarding the overall quality classification, most studies were classified as low (24/62) or medium (28/62) quality. Only 10 studies were classified as high quality. None of the studies met all quality criteria, but five studies met all except one criterion.

### Intervention Effectiveness

**Fig. 3:**
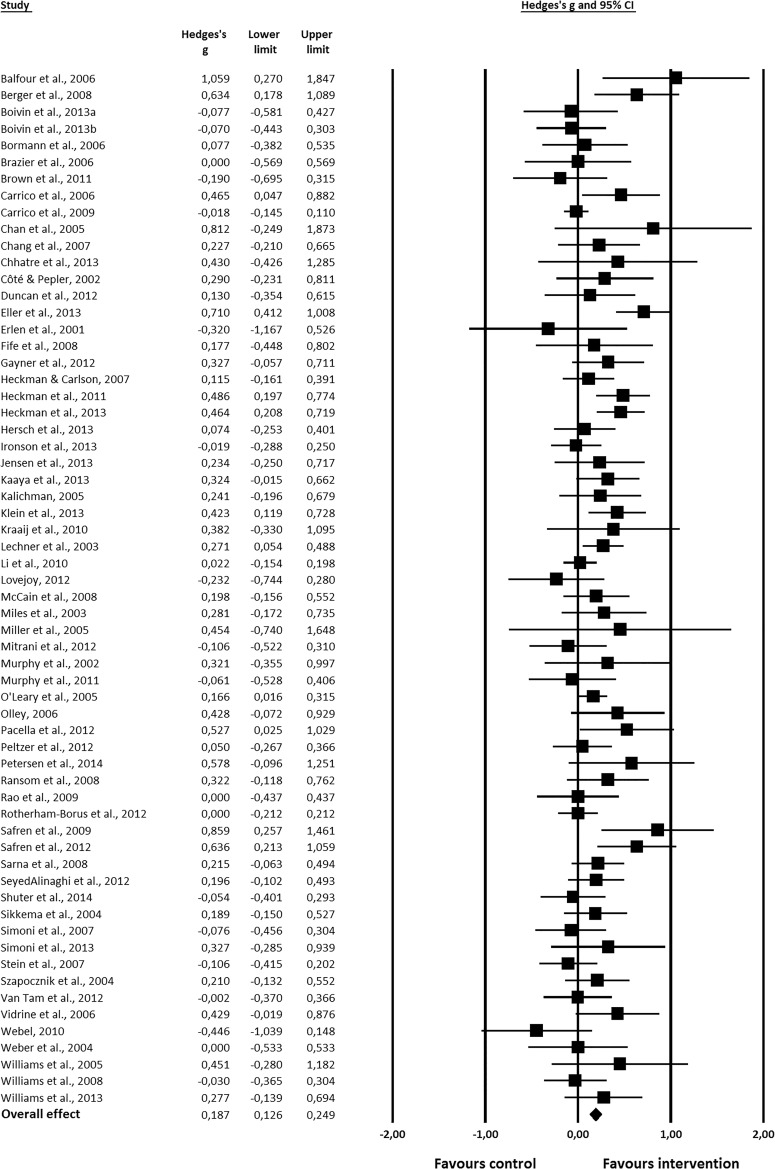
Forest plot showing the effect of psychosocial interventions on mental health outcomes

The overall effect of psychosocial interventions on mental health outcomes was Hedges’ *ĝ* = 0.19, 95% CI [0.13, 0.25], *p* < 0.001 (see Fig. 3). Thus, psychosocial interventions may have a positive effect on mental health, compared to a control condition. However, the effect size was small. Standardized residuals were inspected to identify outliers (studies with standardized residuals |3|), but none were found.

Table 4 shows the overall effect of psychosocial interventions on mental health and the effect sizes for each outcome, intervention type and time point separately. It shows that the pooled effect sizes for depression and psychological well-being were larger (*ĝ* = 0.21 and 0.20) than those for anxiety and quality of life (*ĝ* = 0.09 and 0.13). Furthermore, heterogeneity was moderate and significant for the outcomes depression and psychological well-being and smaller and not significant for anxiety and quality of life. Therefore, we decided to conduct the moderator analysis only on the outcomes depression and psychological well-being. Results are presented for the moderator analyses on depression and differences with the moderator analyses of psychological well-being will be discussed.

**Table 4:** Overall analysis and analysis separately for each outcome, intervention type and time point

Analysis	Subgroup	*k* ^a^	Hedges’ *ĝ*	95% CI	*Q* ^b^	*I* ^2^ (%)^c^
Overall effect		62	0.19^d^	0.13, 0.25	99.35^d^	39
Outcome	Depression	47	0.21^d^	0.13, 0.29	87.32^d^	47
	Anxiety	22	0.09	−0.01, 0.19	31.29	33
	Quality of life	19	0.13^d^	0.04, 0.21	21.07	15
	Psychological well-being	25	0.20^d^	0.09, 0.31	44.63^d^	46
Intervention type	Symptom-oriented intervention	39	0.19^d^	0.11, 0.28	69.71^d^	46
	Supportive intervention	20	0.21^d^	0.09, 0.33	43.58^d^	56
	Meditation intervention	9	0.20^d^	0.06, 0.35	2.00	0
Time point	Posttest 0–3 months	59	0.18^d^	0.12, 0.25	85.79^e^	32
	Posttest 3–6 months	19	0.13^d^	0.05, 0.22	28.31	36
	Posttest 6–9 months	12	0.18^d^	0.05, 0.31	22.97^e^	52
	Posttest >9 months	9	0.08	−0.05, 0.21	13.79	42

^a^ *k* = number of studies

^b^ *Q* = heterogeneity test

^c^ *I* ^2^ = % of heterogeneity

^d^ *p* < 0.01

^e^ *p* < 0.05

The results regarding intervention type (categories: symptom-oriented intervention, supportive intervention, and meditation intervention; Table 4) show that each intervention type had a pooled effect size of approximately *ĝ* = 0.20. The analysis on time points shows that the first and third time point had pooled effect sizes of *ĝ* = 0.18, while the second (*ĝ* = 0.13) and last (*ĝ* = 0.08) time point had smaller pooled effect sizes. The pooled effect size of the last time point was not significant and it should be noted that it was based on only nine studies. In sum, the overall effect of psychosocial interventions on mental health outcomes was small (*ĝ* = 0.19).

### Intervention Effectiveness: Analysis on Last Time Point

The analysis on outcome type and intervention type described above was also conducted with the last available time point for each study, instead of the first time point. The analysis on the last time point was conducted, as we were also interested in the results on the long term, next to the results on the short term. Differences between those analyses were examined and will be depicted here. The overall effect of psychosocial interventions on mental health was comparable, *ĝ* = 0.18, 95% CI [0.12, 0.25], *p* < 0.001; *Q* = 110.25, *p* < 0.001, *I* ^2^ = 45%. The pooled effect size on the outcome of anxiety was somewhat larger in this analysis, *ĝ* = 0.14, 95% CI [0.02, 0.25], *p* < 0.05 and heterogeneity was significant, *Q* = 39.44, *p* < 0.01, *I* ^2^ = 47%. The pooled effect size on the outcome psychological well-being was comparable, but heterogeneity was smaller and not significant in this analysis, *Q* = 34.06, *p* = 0.08, *I* ^2^ = 30%. Furthermore, the effects of supportive interventions (*ĝ* = 0.18, 95% CI [0.04, 0.33], *p* < 0.05) and meditation (*ĝ* = 0.16, 95% CI [0.02, 0.31], *p* < 0.05) were somewhat smaller in this analysis and the effect of symptom-oriented interventions was larger (*ĝ* = 0.21, 95% CI [0.14, 0.28], *p* < 0.001). Summarizing, the analysis on the first time point and the analysis on the last time point were comparable and only small differences were found.

### Publication Bias

**Fig. 4:**
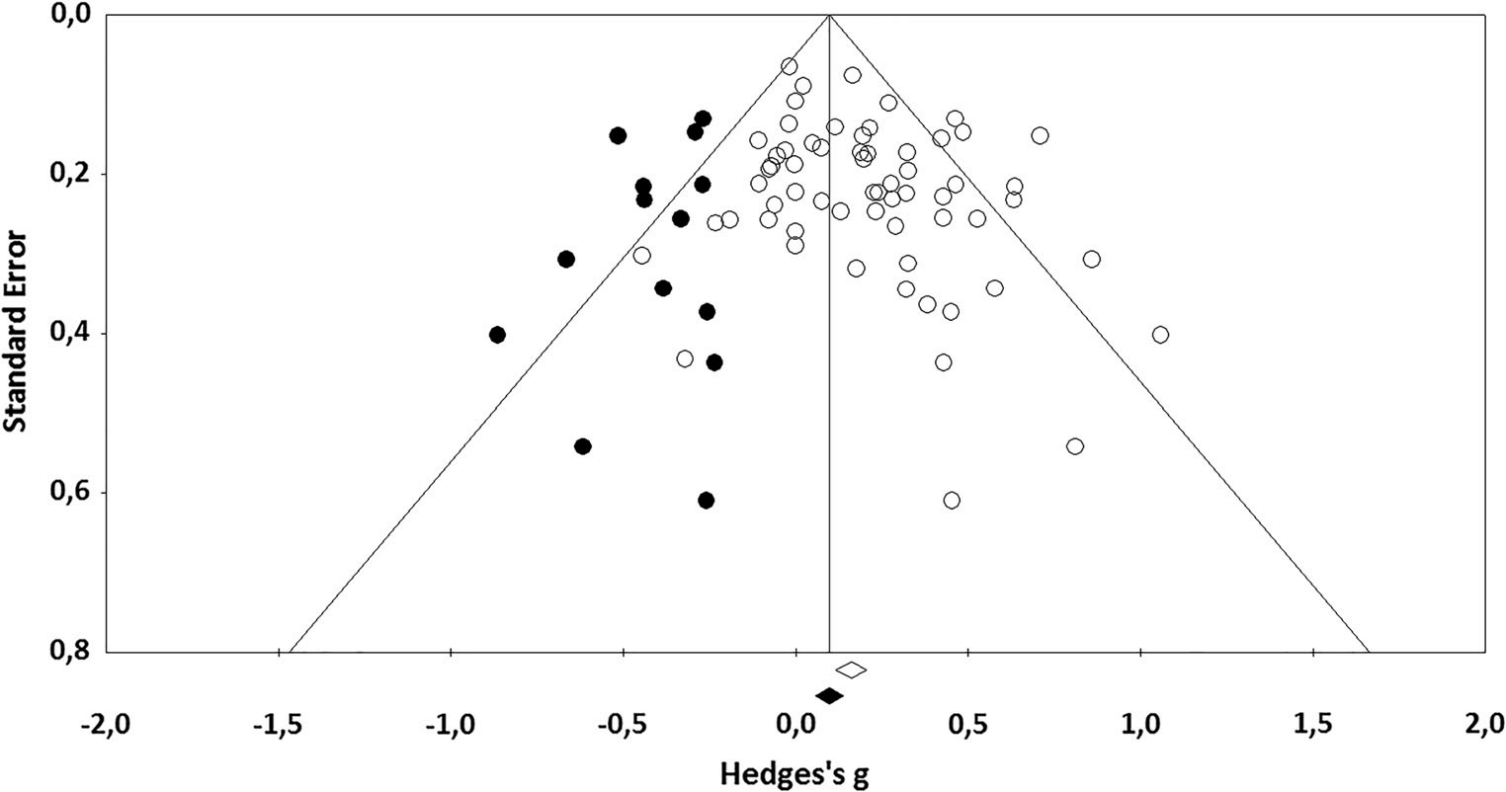
Funnel plot of standard error by Hedges’s *g* with imputed studies from Duval and Tweedie’s trim and fill analysis

When the funnel plot was inspected (see Fig. 4), it was clear that studies were missing on the left side of the plot. This may be an indication of publication bias. Egger’s test of the intercept was significant, intercept 0.82, 95% CI [0.09, 1.54], *t*(60) = 2.24, *p* < 0.05. This also indicates that there may be publication bias. Lastly, Duval and Tweedie’s trim and fill analysis demonstrated that 14 studies were missing on the left side of the plot (see black dots in Fig. 4). After imputation of these 14 studies, the adjusted effect size was *ĝ* = 0.11, 95% CI [0.04, 0.17]. This effect size is substantially smaller than the unadjusted effect size of *ĝ* = 0.19. In sum, there seems to be evidence for publication bias in this meta-analysis, as studies with smaller effect sizes are missing.

### Moderator Analysis on the Outcome Depression

Table 5 shows the effects of the subgroup analysis on the outcome depression. It shows that the type of control group was a significant moderator. Contrary to expectations, studies that used a waiting list control group had smaller effect sizes in general, than studies that used an active or standard care control condition. However, there were only five studies in this analysis that used a waiting list control group. Also, the percentage of people with AIDS in a study was a significant moderator, i.e., when less than 40% of the participants in the studies had AIDS the effect sizes were on average larger than when 40% or more of the participants in the studies had AIDS. Furthermore, the moderator a priori screening on depression was significant, which means that studies that only included participants with depressive symptoms had larger effect sizes in general (*ĝ* = 0.46, 95% CI [0.25, 0.68]), than studies that did not had the presence of depressive symptoms as an inclusion criterion (*ĝ* = 0.12, 95% CI [0.05, 0.18]). Last, the moderator provider of the intervention was significant. Studies that had a psychologist or a psychotherapist as a provider of the intervention had the largest pooled effect sizes. Studies that used other providers (e.g., a counsellor or peer or a computer) had smaller pooled effect sizes. Concluding, the moderators that were found to be significant were: type of control group, percentage of people with AIDS, a priori screening on depression, and provider of the intervention.

**Table 5:** Moderators of intervention effect on depression (k = 47)

Moderator	Subgroup	*k* ^a^	Hedges’ *ĝ*	95% CI	*Q* for difference^b^
Control group	Active control group	24	0.22^c^	0.11, 0.33	6.74^d^
	Standard care	18	0.25^c^	0.14, 0.36	
	Waiting list	5	−0.04	−0.24, 0.16	
Location	Africa/Asia	8	0.19^c^	0.05, 0.33	0.01
	North America/Europe	38	0.19^c^	0.10, 0.27	
	Other^e^	1			
First year recruitment	1996–2001	16	0.13^d^	0.02, 0.24	4.37
	2002–2006	15	0.32^c^	0.16, 0.47	
	2007–2012	13	0.13	−0.01, 0.28	
	Missing	3			
% Attrition	0–10%	14	0.18^d^	0.01, 0.36	1.14
	10–20%	17	0.20^c^	0.08, 0.32	
	>20%	14	0.28^c^	0.15, 0.41	
	Missing	2	0.03		
% Females	0–20%	14	0.27^c^	0.15, 0.40	4.54
	20–80%	22	0.21^c^	0.09, 0.33	
	80–100%	10	0.08	−0.06, 0.21	
	Missing	1			
Mean age	<42.40 years	19	0.14^c^	0.04, 0.24	0.74
	≥42.40 years	22	0.21^c^	0.09, 0.32	
	Missing	6			
% MSM^f^	0%	10	0.10	−0.05, 0.24	0.50
	>0%	10	0.17^d^	0.02, 0.31	
	Missing	27			
% Participants with AIDS	<40%	5	0.54^c^	0.38, 0.71	7.24^c^
	≥40%	5	0.19	−0.01, 0.38	
	Missing	37			
Mean duration HIV	<10.02 years	10	0.16	−0.05, 0.38	0.21
	≥10.02 years	13	0.22^c^	0.07, 0.38	
	Missing	24			
% on ART^g^	<87%	10	0.34^c^	0.15, 0.52	0.00
	≥87%	12	0.34^c^	0.18, 0.49	
	Missing	25			
Screening on depression	No	37	0.12^c^	0.05, 0.18	9.13^c^
	Yes	10	0.46^c^	0.25, 0.68	
Mental health primary outcome	No	6	0.17	−0.02, 0.36	0.19
	Yes	41	0.22^c^	0.13, 0.30	
Provider intervention	Psychologist	13	0.42^c^	0.28, 0.56	12.92^d^
	Counsellor	21	0.15^c^	0.05, 0.25	
	Peer	2	0.10	−0.04, 0.25	
	None	5	0.18	−0.19, 0.54	
	Other (practitioner)	1	0.06	−0.22, 0.33	
	Missing	5			
Format intervention	Group	19	0.23^c^	0.14, 0.33	4.58
	Individual	20	0.25^c^	0.10, 0.40	
	Combination	4	0.09	−0.16, 0.33	
	Other	4	−0.01	−0.26, 0.23	
Analysis	ITT^h^	24	0.22^c^	0.11, 0.34	1.32
	No ITT^h^	15	0.13^d^	0.02, 0.24	
	Missing	8			
Study quality	Low	17	0.23^c^	0.08, 0.38	0.53
	Medium	24	0.19^c^	0.09, 0.29	
	High	6	0.28^d^	0.001, 0.57	

^a^ *k* = number of studies

^b^ *Q* = *Q* for difference between subgroups

^c^ *p* < 0.01

^d^ *p* < 0.05

^e^One study recruited participants in South Africa, Puerto Rico and the USA. This study could not be classified into one of the categories, so it was removed from the moderator analysis on location

^f^ *MSM* men that have sex with men

^g^ *ART* antiretroviral therapy

^h^ *ITT* intent to treat analysis

### Moderator Analysis: Effect of Intervention Characteristics on the Outcome Depression

Table 6 shows the effects of intervention characteristics on the outcome of depression. In this analysis, all interventions were investigated separately, so some studies are represented twice. When mental health (i.e., depression, anxiety, quality of life, or psychological well-being) was a primary focus of the intervention in a study, the effect sizes were in general larger than when mental health was not a primary focus of an intervention. Furthermore, treatment duration was a significant moderator. Studies with treatment durations of 12–18 h had the largest effect sizes. Studies with shorter or longer treatment durations had smaller effect sizes in general. In sum, the following moderators were significant in this analysis: mental health primary focus of the intervention and treatment duration.

**Table 6:** Effect of intervention characteristics on depression

Moderator	Subgroup	*m* ^a^	Hedges’ *ĝ*	95% CI	*Q* for difference^b^
Relaxation technique^c^	No	22	0.18^d^	0.06, 0.29	2.75
	Yes	9	0.38^d^	0.17, 0.59	
Cognitive behavioral technique^c^	No	9	0.15	−0.07, 0.38	0.53
	Yes	22	0.25^d^	0.13, 0.37	
Motivational interviewing technique^c^	No	25	0.23^d^	0.12, 0.34	0.07
	Yes	6	0.18	−0.16, 0.52	
Stress-management technique^c^	No	20	0.19^d^	0.06, 0.31	1.04
	Yes	11	0.29^d^	0.13, 0.45	
Mental health primary focus intervention	No	22	0.14^d^	0.06, 0.22	4.02^e^
	Yes	31	0.29^d^	0.02, 0.41	
Theory-driven intervention	No	19	0.22^d^	0.09, 0.36	0.01
	Yes	34	0.21^d^	0.12, 0.31	
Treatment duration	1–5 h	13	0.06	−0.09, 0.20	10.76^e^
	5–12 h	14	0.12	−0.01, 0.25	
	12–18 h	11	0.38^d^	0.23, 0.53	
	18–30 h	9	0.17	−0.003, 0.33	
	Missing	6			

^a^ *m* = number of interventions

^b^ *Q* = *Q* for difference between subgroups

^c^The effect of the use of specific intervention techniques was investigated in symptom-oriented interventions only (*m* = 31)

^d^ *p* < 0.01

^e^ *p* < 0.05

### Moderator Analysis on the Outcome Psychological Well-Being

The moderator analysis was also conducted on the outcome psychological well-being, next to the moderator analysis on the outcome depression. It was decided to do the moderator analysis on these two outcomes, because the largest pooled effect sizes were found for depression and psychological well-being and heterogeneity was highest and significant for these outcomes (see Table 4). Differences between the moderator analysis on the outcome depression and psychological well-being will be discussed here. The moderators percentage of people with AIDS, provider of the intervention, whether mental health was a primary focus of the intervention and treatment duration were not significant in the analysis on well-being, all *p*’s > 0.06. The type of control group remained a significant moderator. The moderator screening on the presence of depressive symptoms was not included in this analysis, since the outcome was psychological well-being and therefore most studies did not screen on depressive symptoms in these studies. Furthermore, the percentage of people on ART (*Q* = 4.10, *p* < 0.05) and study quality (*Q* = 8.71, *p* < 0.05) were significant moderators in this analysis. Regarding the percentage of people on ART, the largest effect sizes were in general for studies with 87% or more of the participants on ART (*ĝ* = 0.39, 95% CI [0.14, 0.64], *p* < 0.01, *k* = 8), and studies with less than 87% of participants on ART had smaller effect sizes (*ĝ* = 0.07, 95% CI [−0.11, 0.25], *p* = 0.45, *k* = 3). Though, the last category contained only three studies. Regarding study quality, studies with a medium quality had larger effect sizes on average (*ĝ* = 0.30, 95% CI [0.14, 0.46], *p* < 0.001, *k* = 14), than studies with a low (*ĝ* = 0.15, 95% CI [−0.05, 0.35], *p* = 0.15, *k* = 7) or high quality (*ĝ* = −0.04, 95% CI [−0.21, 0.12], *p* = 0.61, *k* = 4). However, there were only four studies in the category high quality in this analysis. Summarizing, in the moderator analysis on the outcome psychological well-being the significant moderators were: type of control group, percentage of people on ART, and study quality.

## Discussion

The first aim of this systematic review and meta-analysis was to investigate the effectiveness for PLWH of various psychosocial interventions aimed at decreasing depression and anxiety, and improving quality of life and psychological well-being, and to investigate which interventions were the most effective on these outcomes. Sixty-two studies were included in the analysis, and it was found that psychosocial interventions had a positive effect on the mental health outcomes described, although the effect size was small. In addition, there was evidence of publication bias, so the corrected effect size was smaller. Most studies in the meta-analysis were categorized as low or medium quality studies, there was a lack of high quality studies. Furthermore, a range of psychosocial intervention types can be effective for PLWH, from symptom-oriented interventions such as CBT, to supportive interventions and meditation. There were no differences in effectiveness between these different intervention types, so they all seem to be helpful in improving the mental health of PLWH.

The second aim of the current meta-analysis was to study moderators of intervention effect, to determine whether important characteristics of a study or a therapy may influence the effectiveness of the treatments in question. We found that six factors may influence the effectiveness of a treatment for depression. Of these six factors, three could be classified as intervention characteristics: who provided the intervention, whether mental health was a primary focus of the intervention, and what the duration of treatment was. The other three were study characteristics: whether there was a priori screening for depression, what percentage of the participants in a study had AIDS, and what type of control group was included. Other factors, such as intervention techniques, were shown not to moderate intervention effect.

We found that, overall, psychosocial interventions had a positive effect on depression, anxiety, quality of life, and psychological well-being of PLWH. However, the pooled effect size was small, *ĝ* = 0.19. When the pooled effect sizes of the separate outcomes were examined, it was found that the pooled effect sizes on depression and psychological well-being were the largest; smaller pooled effect sizes were found on anxiety and quality of life. Previous meta-analyses [14–16] found mostly small to moderate effect sizes of psychosocial interventions for PLWH for the outcomes depression and anxiety. The effect size on the outcome quality of life was comparable with a previous meta-analysis, which also found a small effect [16]. Furthermore, two previous meta-analyses investigated the effects of CBT and stress-management on stress (stress was included in the outcome psychological well-being in the present meta-analysis), and one of these found a moderate [14] and the other a small pooled effect size [16]. These differences in effect sizes between the current study and previous ones may be explained by a difference in the focus of the interventions included. When the moderator analysis was conducted, it was found that when mental health was the primary focus of an intervention, the effects were larger than when this was not the primary focus of an intervention. Previous meta-analyses mainly included interventions whose primary aim was to improve mental health. This important difference between the current meta-analysis and previous meta-analyses may explain the smaller effects in this study. In addition, the current meta-analysis included 62 studies, while most previous meta-analyses included less than half this number of studies. The inclusion criteria of this meta-analysis were also broader: various psychosocial interventions and outcomes were included. For these reasons, this meta-analysis may have more power to detect a true effect.

Furthermore, the analysis was conducted at several time points, to study the effect of interventions in both the short and the long term. It was found that the effect size was much smaller at the last time point (9 months or more after the intervention had ended), than at the earlier time points. Two previous meta-analyses [14, 18] about the effectiveness of CBT for PLWH with mental health problems also found that the effects were smaller on later follow-up assessments. This may indicate that the positive effects of interventions on the mental health of PLWH may wear off after a while. Booster sessions, follow-up sessions after termination of the therapy to prevent relapse, could be helpful to retain the effects. A similar finding emerged in a meta-analysis about the long-term effects of psychotherapy for depression [94]. However, only nine studies in our meta-analysis had data available on the last time point, so the results should be interpreted with caution. For future studies into psychosocial interventions for PLWH with mental health problems, we advise including a longer follow-up period to further investigate the long-term effects. In addition, future research could study the effect of booster sessions.

In the analysis on intervention types, no differences were found between the various intervention types. This is in line with previous meta-analyses about psychological therapies for depression in the general population or in people with medical disorders, which also found no differences in effectiveness between interventions such as CBT, interpersonal therapy, supportive therapy, and problem-solving therapy [94–98]. It seems that several types of interventions may be useful to improve the mental health of PLWH. It has previously been argued more generally that various forms of psychotherapy may have the same effect, because they share common factors, such as the relationship with the therapist [99, 100]. The specific type of therapy does not seem to be that important. This corresponds with our findings. For a more thorough investigation of the differences in effect between psychosocial interventions for PLWH, it is important to design studies that compare different types of interventions. Furthermore, it would also be interesting to know more about how treatments work (mediating factors), and to compare this between different treatments. Future studies should focus on these topics.

In addition to the analysis on intervention types, we also investigated differences in effect between intervention techniques in the symptom-oriented interventions. No differences in effect were found between symptom-oriented interventions that included techniques of relaxation, CBT, stress-management, or motivational interviewing, on the one hand, and symptom-oriented interventions that did not include these techniques, on the other. This is related to the findings about intervention types and a previous meta-analysis that also did not find any differences between interventions that included or did not include stress management skills training [14].

The subgroup analyses indicated that there were several moderators that influenced the effects of interventions on depression. An important moderator was a priori screening for depressive symptoms: when studies included only participants with depressive symptoms the effect sizes were larger than when the presence of depressive symptoms did not serve as an inclusion criterion. This result seems evident: there is more to gain for PLWH with depressive symptoms than for PLWH without depressive symptoms. A previous meta-analysis also found that in studies that included PLWH with more anxiety symptoms at baseline, the participants benefited more from stress-management interventions [16]. On the other hand, another meta-analysis into the effectiveness of CBT for PLWH with depression and anxiety found no inclusion-related differences between studies: there was no difference between studies that restricted participation to those with depressive symptoms and studies that did not have the presence of depressive symptoms as an inclusion criterion [14]. However, in this meta-analysis the number of studies in each category was low. All in all, when an intervention is aimed at reducing depression, it seems favorable to screen PLWH a priori and only offer them the treatment if they actually suffer from depressive symptoms.

Another significant moderator was whether mental health was the primary focus of an intervention. In studies where mental health was the primary focus of the intervention, the effects were larger than in studies where mental health was not the primary focus. Again, this result seems logical: if the aim of an intervention is to reduce depression, participants will work on reducing symptoms during treatment, and it is expected that this will be effective. If the aim of an intervention is to quit smoking, for example, this will be the focus of the therapy and it is not so likely that participants’ psychological symptoms will also improve.

We found that studies that had a psychologist or psychotherapist as a provider of the intervention had the largest pooled effect sizes. Studies that had other providers, e.g., counsellors, peers, or computers, all had smaller effect sizes in general. This shows that it may not be so important which specific therapy or technique is used to treat mental health problems in PLWH; the key element may be the provider of the intervention. Psychologists have a broad training in treating mental health problems, so they may be more experienced and more competent to help PLWH in need. This is in contrast with most other providers; they may be trained to provide the intervention, but this may not be comparable to psychologists’ education and experience in mental health care. However, a previous meta-analysis about CBT for depression and anxiety in PLWH found no differences in effects between studies in which interventions were provided by a psychologist or psychiatrist and studies in which interventions were delivered by trained research staff (e.g., graduate- and doctoral-level students) [14]. Further, two meta-analyses on guided self-help [101] or guided computerized interventions [102] for depression or anxiety also did not find differences in effects between studies involving experienced providers (e.g., psychologists) and those involving less experienced providers (e.g., students). However, the moderator analysis in this meta-analysis comprised many more studies than those in the previous meta-analyses, so it has more power to detect differences. To conclude, psychologists and psychotherapists may be the most qualified providers of psychosocial treatments for PLWH with mental health problems. More research is needed to confirm this.

The duration of treatment was another important moderator in this meta-analysis. We found that studies with a treatment duration of 12–18 h had the largest effect sizes, compared to treatments of shorter or longer duration. So, it seems that therapies of average duration may be more effective than treatments of short or long duration. However, there is a trend toward designing concise treatments for mental health problems, which can be provided via the Internet and are thought to be more cost-effective. An RCT that compared the effectiveness of concise CBT with standard CBT for depression and anxiety found that they were equivalent [103]. Most previous meta-analyses that have investigated the effectiveness of psychological interventions for depression or anxiety have also found no differences between treatments with a short or long duration [14, 95, 97, 101, 102]. One meta-analysis about online CBT for patients with chronic somatic conditions and depression did find an effect of treatment duration, with a larger effect size for treatments with a longer duration [104]. Some of the previous meta-analyses [14, 101, 102, 104] included a small number of studies in the moderator analysis, but other meta-analyses [95, 97] included more than 100 studies. Hence, it is not yet clear whether the effectiveness of a treatment is related to its duration. When comparing short and long treatments for mental health problems, it may be useful to take the severity of the symptoms into account. People with more severe symptoms may need more sessions than people with a mild or moderate symptom severity [105]. Future studies may focus on this topic.

Contrary to expectations, the type of control group was a significant moderator in this meta-analysis. Studies that had a waiting list control group had smaller effect sizes than studies with a standard care or active control group. This is counterintuitive, since participants on a waiting list do not receive any treatment, which would lead us to expect large differences between the intervention and the control condition [106]. However, there were only five studies in the moderator analysis that used a waiting list control condition, so firm conclusions cannot be drawn. Previous meta-analyses on the effectiveness of interventions for depression and anxiety found no differences between control group types [14, 94, 102], or found that the studies that used a waiting list control condition had larger effect sizes than other control group types [95, 101, 102].

The last significant moderator was the percentage of participants in the study who had AIDS. When fewer than 40% of the participants in a study had received a diagnosis of AIDS, the effect sizes were larger than when more than 40% of the participants had AIDS. People with AIDS are generally more physically ill—they suffer from more pain and lack of energy—than people without AIDS. This physical discomfort may have a great influence on their mental well-being: i.e., they may feel more sad, worry more, or have difficulties sleeping [1]. Therefore, it may be more difficult to treat these psychological symptoms in people with AIDS. That is, the physical symptoms remain, and their influence on the mental state may hamper a successful response to treatment. Therefore, it may be important to combine medical and psychosocial treatments in people with AIDS, to try to improve or stabilize both the physical and psychological symptoms. In this situation, effective collaboration between treatment providers is crucial. It should be noted that this moderator analysis was based on only ten studies, so the results should be interpreted cautiously and may not be generalizable to other study samples.

This meta-analysis had some limitations, which will be discussed here. First, there was evidence of publication bias. This may indicate that studies with negative effect sizes were missing in the analysis. When these possible missing studies were imputed and a corrected effect size was calculated, it was smaller than the uncorrected effect size. So, it has to be concluded that the overall effect of psychosocial interventions on the mental health of PLWH is small. Second, the quality of the studies included was mostly low or medium; only 16% of the studies included was of high quality. The quality criteria regarding the concealment of allocation to conditions and the availability of a published study protocol, especially, were often not clearly reported in the studies included. Therefore, it is possible that some studies were classified as low or medium quality studies now, while they may have been classified as high quality studies when there would be more information in the paper concerning these criteria. Besides this, it is evident that low quality studies often did not do an intent-to-treat analysis. It would be advisable for future studies to state whether they have complied with the criteria, and that incomplete outcome data will be adequately addressed. A limitation of the instruments used to assess study quality is that when many criteria are not clearly described in a paper, a study was classified as a low quality study. However, study quality was not a significant moderator in the analysis on depression, so studies of high quality did not have larger effect sizes than studies of lower quality. Third, in some of the moderator analyses, only a few studies could be included. This is related to the fact that some studies did not report on all moderator variables. Consequently, the results of the moderator analyses with few studies may not be representative for all of the studies included, and the power is lower in these analyses. Fourth, many moderator analyses were performed in this study, and no correction for multiple testing was applied. This increases the risk of finding spurious moderator effects. Fifth, the outcomes in this meta-analysis were restricted, so the effect of psychosocial interventions on other relevant outcomes (e.g., PTSD) was not investigated. Though, a recent review [107] found two CBT-based interventions that were effective in decreasing PTSD symptoms in PLWH. Therefore, it is possible that the findings of the current meta-analysis also apply to PTSD. However, only two studies were found in the review, so more research into interventions for PTSD in PLWH is necessary. Sixth, although we searched in three databases and in the references of previous meta-analyses and reviews, it is still possible that some relevant articles were not found with this search strategy. Seventh, the moderator concerning the theory content of the intervention had two categories: theory driven or not theory-driven. For each study, it was determined to which of the categories it belonged by reading the paper. It could be argued that this is not a completely thorough approach, because an intervention may still be based on theory, despite the fact that it is not stated in the paper. Furthermore, interventions may be evidence-based, but not based on a specific theory. Or the other way around: it may be based on a theory, but it is not evidence-based. So, it is recommended for future studies to mention in the paper whether the investigated intervention was theory-driven and/or evidence-based. Last, in the moderator analysis on the effect of intervention characteristics, all interventions were investigated separately. This approach was chosen because in some studies two interventions were investigated, and the interventions did not always belong to the same category of a moderator (e.g., one intervention in a given study may have a treatment duration of 4 h and another a treatment duration of 8 h). Therefore, six studies were represented twice in these analyses. It would be preferable to use each study just once in the analysis, but this was not possible here.

Some recommendations for future research may be derived from the results of this meta-analysis. First, future studies should focus on investigating differences between various interventions: how they work and for whom they work. Second, the long-term effects of psychosocial interventions and the effect of booster sessions should be investigated more thoroughly in the future. Third, most studies in the current meta-analysis were conducted in the USA and in Europe. Since the prevalence of HIV is high in low and middle income countries and mental health problems are common in this population, interventions to treat these problems are needed [108]. However, there are significant barriers to providing mental health services in these countries, e.g., there is a lack of trained mental health workers [109]. Therefore, it is important that these interventions are adapted to the local culture, are brief, can be provided by non-specialists, and are tailored for PLWH. It was found that interventions for PLWH in low and middle income countries were effective when they were focused on the family and integrated into community based health care [110]. More research is recommended on mental health interventions for PLWH in low and middle income countries. Fourth, there were a lot of changes in the past twenty years in the mental health care for PLWH and in study methodologies. We investigated the effects of many moderators, but there will be issues that were not addressed. For future studies, it is important to be aware of the changes in mental health care and study design. Fifth, it is likely that moderator effects were related, e.g., when there was a screening for depression in a study, this may be related to the fact that the primary focus of the intervention in the study was on mental health. These relations between moderators were not examined in the current meta-analysis, but are interesting to investigate in future meta-analyses. The most optimal result of an intervention may be obtained when the most effective characteristics will be combined in an intervention. Sixth, the moderation analysis indicated that the effect sizes were larger for studies that solely included PLWH with mental health problems and for interventions that were focused on mental health. Therefore, it is recommended in future intervention studies to restrict the inclusion to participants with mental health problems and to design interventions that are focused on improving mental health. Last, for new studies, it is important to measure and report on study and treatment characteristics, so that studies and interventions can be compared in meta-analyses.

To conclude, this systematic review and meta-analysis included 62 RCTs and therefore has high power. In addition, the effects of multiple intervention and study characteristics on treatment outcome were investigated. The meta-analysis found that, overall, psychosocial interventions may have a small positive effect on the mental health of PLWH. No differences in effect were found between the three intervention types, which means that symptom-oriented interventions, supportive interventions, and meditation may all be effective. A larger improvement in depression may be obtained when only participants with depressive symptoms are included in the study; when interventions are provided by psychologists; when treatment duration is 12–18 h; and when the intervention is focused on improving mental health. Based on the results of this meta-analysis, it is important to incorporate psychosocial therapies into the care of PLWH with mental health problems.

## Appendix: Search Strategy

### PubMed Search Term

(hiv [mesh] OR hiv infection [mesh] OR hiv [tiab] or aids [tiab]) AND (psychotherapy [mesh] OR mental health services [mesh] OR self-care [mesh] OR self-help groups [mesh] OR telemedicine [mesh] OR therapy, computer-assisted [mesh] OR psychotherap* [tiab] OR psychological therap* [tiab] OR psychological treatment* [tiab] OR psychological intervention* [tiab] OR counsel* [tiab] OR cbt [tiab] OR behavior therap* [tiab] OR behaviour therap* [tiab] OR interpersonal therap* [tiab] OR coping [tiab] OR peer support [tiab] OR social support [tiab] OR problem solving [tiab] OR stress manage* [tiab] OR self-help [tiab] OR internet therap* [tiab] OR online therap* [tiab] OR psychoed* [tiab] OR training [tiab] OR exposure [tiab] OR relaxation [tiab] OR mindfulness [tiab] OR reinforcement [tiab] OR risk reduction [tiab] OR commitment therap* [tiab] OR case manage* [tiab]) AND (depression [mesh] OR depress* [tiab] OR anxiety [mesh] OR anxi* [tiab] OR fear [tiab] OR quality of life [mesh] OR quality-of-life [tiab] OR well-being [tiab] OR stress, psychological [mesh] OR stress* [tiab] OR distress [tiab] OR mental health [tiab])

*Filters used* controlled clinical trial or randomized controlled trial, publication date from 1996/01/01, humans, English language.

### PsycInfo Search Term

(DE (hiv OR aids) OR TX (hiv OR aids)) AND (DE (psychotherapy OR psychotherapeutic techniques OR mental health programs OR Counseling OR Stress management OR case management OR self management OR Telemedicine OR Computer Assisted Therapy OR Psychoeducation) OR TX (psychotherap* OR psychological-therap* OR psychological-treatment OR psychological-intervention OR counsel* OR cbt OR behavio#r-therap* OR interpersonal-therap* OR coping OR peer-support OR social-support OR problem-solving OR stress-manage* OR self-help OR internet-therap* OR online-therap* OR psychoed* OR training OR exposure OR relaxation OR mindfulness OR reinforcement OR risk-reduction OR commitment-therap* OR case-manage*)) AND (DE (major depression OR anxiety OR stress OR distress OR quality of life OR well being) OR TX(depress* OR anxi* OR fear OR quality-of-life OR well-being OR stress* OR distress OR mental-health))

*Filters used* publication year 1996–2015, adulthood (18 years and older), experimental replication or treatment outcome/clinical trial or follow-up study.

### Embase Search Term

(exp “Human immunodeficiency virus”/OR exp “Human immunodeficiency virus infection”/OR exp “Acquired immune deficiency syndrome”/OR hiv.tw. OR aids.tw.) AND (exp psychotherapy/or exp “mental health services”/or exp “self care”/OR exp “self help”/OR exp teletherapy/OR exp “computer assisted therapy”/OR psychotherap*.tw. OR psychological-therapy.tw. OR psychological-treatment.tw. OR psychological-intervention.tw. OR counsel*.tw. OR cbt.tw. OR behavio?r-therapy.tw. OR interpersonal-therapy.tw. OR coping.tw. OR peer-support.tw. OR social-support.tw. OR problem-solving.tw. OR stress-management.tw. OR self-help.tw. OR internet-therap*.tw. OR online-therap*.tw. OR psychoed*.tw. OR training.tw. OR exposure.tw. OR relaxation.tw. OR mindfulness.tw. OR reinforcement.tw. OR risk-reduction.tw. OR commitment-therap*.tw. OR case-manage*.tw.) AND (exp depression/OR depress*.tw. OR exp anxiety/OR anxi*.tw. OR exp fear/OR fear.tw. OR “quality of life”/exp OR quality-of-life.tw. OR well-being.tw. OR exp stress/OR “mental stress”/exp OR stress*.tw. OR distress.tw. OR mental-health.tw.)

*Filters used* human, English language, records from Embase, randomized controlled trial or controlled clinical trial, publication year 1996–2015, article and adult (18–64 years) or aged (65+ years).
